# Preexisting Type 1 Diabetes Mellitus Blunts the Development of Posttraumatic Osteoarthritis

**DOI:** 10.1002/jbm4.10625

**Published:** 2022-04-19

**Authors:** Naiomy D Rios‐Arce, Deepa K Murugesh, Nicholas R Hum, Aimy Sebastian, Elias H Jbeily, Blaine A Christiansen, Gabriela G Loots

**Affiliations:** ^1^ Physical and Life Sciences Directorate Lawrence Livermore National Laboratory Livermore CA USA; ^2^ Department of Orthopedic Surgery UC Davis Medical Center Sacramento CA USA; ^3^ Molecular and Cell Biology School of Natural Sciences, UC Merced Merced CA USA

**Keywords:** CARTILAGE, DIABETES, GENE EXPRESSION PROFILE, OSTEOARTHRITIS, POSTTRAUMATIC OSTEOARTHRITIS

## Abstract

Type 1 diabetes mellitus (T1DM) affects 9.5% of the population. T1DM is characterized by severe insulin deficiency that causes hyperglycemia and leads to several systemic effects. T1DM has been suggested as a risk factor for articular cartilage damage and loss, which could expedite the development of osteoarthritis (OA). OA represents a major public health challenge by affecting 300 million people globally, yet very little is known about the correlation between T1DM and OA. In addition, current studies that have looked at the interaction between diabetes mellitus and OA have reported conflicting results with some suggesting a positive correlation whereas others did not. In this study, we aimed to evaluate whether T1DM exacerbates the development of spontaneous OA or accelerates the progression of posttraumatic osteoarthritis (PTOA) after joint injury. Histological evaluation of T1DM and control joints determined that T1DM mice displayed cartilage degeneration measurements consistent with mild OA phenotypes. RNA sequencing analyses identified significantly upregulated genes in T1DM corresponding to matrix‐degrading enzymes known to promote cartilage matrix degradation, suggesting a role of these enzymes in OA development. Next, we assessed whether preexisting T1DM influences PTOA development subsequent to trauma. At 6 weeks post‐injury, T1DM injured joints displayed significantly less cartilage damage and joint degeneration than injured non‐diabetic joints, suggesting a significant delay in PTOA disease progression. At the single‐cell resolution, we identified increased number of cells expressing the chondrocyte markers *Col2a1*, *Acan*, and *Cytl1* in the T1DM injured group. Our findings demonstrate that T1DM can be a risk factor for OA but not for PTOA. This study provides the first account of single‐cell resolution related to T1DM and the risk for OA and PTOA. © 2022 The Authors. *JBMR Plus* published by Wiley Periodicals LLC on behalf of American Society for Bone and Mineral Research.

## 1 Introduction

Diabetes mellitus is one of the most common metabolic diseases worldwide. According to the National Diabetes Statistics Report published in 2020 (https://www.cdc.gov/diabetes/library/features/diabetes-stat-report.html), in the US, diabetes affects more than 34.2 million people annually, a number that is rising at alarming rates. There are two forms of diabetes mellitus: type 1 diabetes (T1DM) and type 2 diabetes (T2DM). During T1DM, the ability of the pancreatic beta cells to produce insulin is disrupted, leading to impaired glucose uptake by the tissues and an increase in blood glucose. Most T1DM cases are diagnosed during childhood, and it has a strong genetic contribution. On the other hand, T2DM is the most common type of diabetes that mostly develops in adults and is characterized by insulin resistance.^(^ ^1^ ^)^ Well‐known systemic effects of diabetes mellitus include neuropathy, nephropathy, and retinopathy. In addition, most recent studies have also shown an effect of diabetes mellitus on bone and cartilage, a field that is less studied.^(^ ^2^, ^3^, ^4^, ^5^, ^6^, ^7^ ^)^

Osteoarthritis (OA) is a degenerative joint disease that affects at least 32.5 million adults in the US.^(^ ^8^ ^)^ OA is characterized by progressive cartilage loss, increase in bone remodeling, and synovial inflammation.^(^ ^9^ ^)^ There are two types of OA: primary and secondary. Primary OA develops idiopathically during aging and is caused by a combination of genetics and lifestyle factors; secondary OA is most commonly triggered by a traumatic joint injury that exacerbates joint degeneration and cartilage breakdown. It is estimated that at least 50% of people that had a joint injury will develop posttraumatic osteoarthritis (PTOA) within 1 to 2 decades after the injury.^(^ ^10^ ^)^ OA can affect any joint, however, the most common joints affected are the hands, lower back, neck, and weight‐bearing joints such as knees, hips, and feet.^(^ ^8^ ^)^ Currently, there are no approved therapies available to prevent OA or PTOA and existing treatments mainly target OA symptoms such as pain.

To date, several studies have assessed the association between diabetes and OA. However, these studies have reported conflicting results with some suggesting a positive correlation(3, 4 ^)^ whereas others did not.^(^ ^5^, ^6^, ^7^, ^11^ ^)^ Furthermore, some of these studies did not account for the difference in disease progression between T1DM and T2DM, a controversy that can explain the discrepancy in past results. It has been reported that T2DM patients are more prone to develop OA,^(^ ^6^, ^12^ ^)^ and studies in different diabetic animal models also support these results. However, most of these studies did not account for or report body weight changes, a well‐known risk factor for OA.^(^ ^13^, ^14^, ^15^ ^)^ On the other hand, a more recent meta‐analysis did not support the idea that diabetes is an independent risk factor for OA and suggested that an increase in body weight, an effect found more often in T2DM patients, is the main driver of OA in this population.^(^ ^11^ ^)^ Few additional studies are also in support of this hypothesis.^(^ ^5^, ^6^, ^7^ ^)^ Because of the discrepancy in the current literature, an adequate understanding of the association between diabetes and primary and secondary OA becomes a necessary task in the musculoskeletal field.

In the present study, we focused on a well‐characterized T1DM mouse model and investigated whether T1DM is a risk factor for OA and/or PTOA. To assess the role of T1DM in promoting OA, we used the streptozotocin (STZ)‐induced diabetic model.^2^, ^16^^)^ Sixteen‐week‐old control and T1DM male mice were histologically assessed for OA development. T1DM mice exhibited mild OA‐like structural impairments, including modest proteoglycan loss, thinning of the cartilage layer, and clefting of the articular surface when compared with control mice. Whole knee joint RNA sequencing (RNA‐Seq) analysis showed high expression of several matrix‐degrading enzymes in the T1DM group, suggesting a role of these enzymes in contributing to the OA phenotypes. To further determine if OA progression is accelerated after a joint injury in T1DM mice, we used a noninvasive tibial compression injury model. Our findings show that despite the mild OA phenotype exhibited by T1DM mice, preexisting T1DM did not accelerate the progression of PTOA. Instead, we found that T1DM mice developed significantly milder PTOA phenotypes after joint injury compared with non‐diabetic injured mice. At single‐cell resolution, we identified significantly more chondrocytes present in the articular cartilage of injured T1DM mice relative to injured non‐diabetic mice. Our findings show that T1DM can be a moderate risk factor for OA but does not accelerate PTOA after joint injury. We also found that the protective effect on the injured T1DM group seems to be driven by preserving the number of *Col2a1‐*, *Acan‐*, and *Cytl1*‐expressing chondrocytes in the T1DM group. This study is the first to describe at the single‐cell level T1DM effects on OA and PTOA.

## 2 Materials and Methods

### 2.1 Animals and tibial compression overload injury model

Eight‐week‐old vehicle‐injected control mice (C57BL/6J, stock no. 000664) and STZ‐injected C57BL/6J (stock no. 33853) male mice were purchased from Jackson Laboratory (Bar Harbor, ME, USA). Before arrival at our facilities, 6‐week‐old C57BL/6J male mice received daily intraperitoneal injections of 50 mg/kg STZ for 5 days to induce diabetes. After arrival at our facilities, diabetes status was confirmed by cutting off the tip of the tail and using a blood glucose meter (O'WELL Blood Glucose Monitoring System). Readings of ≥300 mg/dL were used to assign animals to the T1DM groups. For primary OA assessment, joints were harvested at 16 weeks of age. For PTOA assessment, T1DM and control animals were injured at 10 weeks of age using the noninvasive tibial compression overload injury model, as previously described.^(^ ^17^, ^18^ ^)^ Briefly, the mice were anesthetized via isoflurane inhalation and placed in a prone position with right tibias vertically aligned between two platens for tibial compression. The right knee joint was injured using a compressive load of 10 to 12 N. A single noninvasive tibial compression overload at 1 mm/s displacement rate was used to rupture the anterior crucial ligament (ACL) using an electromagnetic material testing system (ElectroForce 3200, TA Instruments, New Castle, DE, USA). Buprenorphine analgesia was administered immediately post‐injury (0.01 mg/kg) for pain relief. Samples were collected 6 weeks post‐injury. All animal experimental procedures were completed in accordance with the Institutional Animal Care and Use Committee (IACUC) guidance at Lawrence Livermore National Laboratory and the University of California, Davis, in AAALAC‐accredited facilities under protocol 278 approved in April 2019.

### 2.2 Histological assessment of disease severity

Injured and contralateral joints from control and T1DM animals were collected at 6 weeks post‐injury or when the mice were 16 weeks old (*n* ≥ 5 per group). Dissected joints were fixed in 4% paraformaldehyde for 72 hours at 4°C, decalcified using 0.5 M EDTA, infiltrated in increasing concentrations of isopropanol, equilibrated into mineral oil, and embedded into paraffin wax. The joints were sectioned in the sagittal plane, and serial medial sections that included the femoral condyles, menisci, and tibial plateaus were cut at 6 μm; stained on glass slides using 0.1% Safranin O (0.1%, Sigma, St. Louis, MO, USA; S8884) and 0.05% Fast Green (0.05%, Sigma; F7252) using standard procedures (IHC World, Woodstock, MD, USA); and imaged using a Leica (Buffalo Grove, IL, USA) DM5000 microscope. Three blind reviewers independently assessed OA severity using modified Osteoarthritis Research Society International (OARSI) scoring parameters for sagittal views;^(^ ^17^, ^20^ ^)^ grade scale: 0–0.5 normal; 1–2 mild; 3–4 moderate; 5–6 severe cartilage damage.

### 2.3 Bulk RNA sequencing and data analysis

Control and T1DM mice were euthanized at 16 weeks old. Knee joints were collected, dissected, and cut at the edges of the joint region with small traces of soft tissue to preserve the articular integrity. Joints were then homogenized in Qiazol (79306, Qiagen, Valencia, CA, USA) using a PRO tissue homogenizer (Bio‐Gen PRO 200, PRO Scientific Inc., Oxford, CT, USA). Total RNA was purified using Rneasy Mini Kit (Qiagen Inc., Germantown, MD, USA) according to the manufacturer's protocol, and the RNA integrity was assessed using a bioanalyzer (Agilent Technologies, Santa Clara, CA, USA). Poly(A)^+^‐enriched cDNA libraries were generated using the Illumina TruSeq RNA Library Prep kit v2 (Illumina Inc., Hayward, CA, USA). The sequencing was performed using an Illumina (Illumina Inc.) NextSeq 500 instrument to generate 75 bp single‐end reads. The quality of sequencing data was checked using FastQC (http://www.bioinformatics.bbsrc.ac.uk/projects/fastqc). Sequence reads were mapped onto the mouse reference genome (mm10) using STAR.^(^ ^21^ ^)^ A matrix of read counts per gene was generated using featureCount. Differentially expressed genes were identified using edgeR.^(^ ^22^ ^)^ Genes with log2‐fold changes >0.5 and false discovery rate (FDR) adjusted *p* value <0.05 were considered as significantly differentially expressed. Ontology analysis was performed using Enrichr.^(^ ^23^ ^)^ RNA data can be obtained through NCBI, accession number GSE198836.

### 2.4 Micro‐computed tomography (μCT) and osteophyte quantification

Injured and contralateral knee joints were dissected and fixed in 4% formaldehyde for 72 hours at 4°C. Samples were then stored in 70% ethanol at 4°C until scanned. Whole knee joints were scanned using a μCT instrument (SCANCO μCT 35, Brüttisellen, Switzerland) according to the rodent bone structure analysis guidelines (X‐ray tube potential = 55 kVp, intensity = 114 μA, 10 μm isotropic nominal voxel size, integration time = 900 ms). Trabecular bone in the distal femoral epiphysis was analyzed by manually drawing contours on 2D transverse slides. The distal femoral epiphysis was designated as the region of trabecular bone enclosed by the growth plate and subchondral cortical bone plate. Epiphyseal trabecular bone volume fraction was determined by quantifying trabecular bone volume per total volume (BV/TV). Trabecular thickness (Tb.Th), trabecular number (Tb.N), and trabecular spacing (Tb.Sp) were also quantified. Mineralized osteophyte volume in injured and contralateral joints was quantified by drawing contours around all heterotrophic mineralized tissue attached to the distal femur and proximal tibia, as well as the whole fabellae, menisci, and patella. Total mineralized osteophyte volume was then determined as the volumetric difference in mineralized tissue between injured and uninjured joints. Statistical analysis was performed using a paired *t* test to compare injured and contralateral knees.

### 2.5 Single‐cell RNA sequencing (scRNA‐Seq) and data analysis

Single‐cell preparation of joint‐derived cells were obtained from freshly dissected 7 days post‐injury and contralateral knee joints from control and T1DM mice, 4 to 5 mice per group. Briefly, the tissue around the joint was removed without disrupting the joint or fracturing the bone and then washed in PBS to further remove superficial cells. Connective tissue was next severed into digestion media 7.5 mL of 3 mg/mL Collagenase type I (Worthington, Lakewood, NJ, USA) with 100 μg/mL Dnase I (Roche, Basel, Switzerland) in DMEM/F12 for two sequential 1‐hour digests at 37°C with shaking at 150 rpm. After each digest, cells were washed in 5 mL PBS with 1% FBS, and supernatants were collected and centrifuged at 500*g*. Red blood cell removal was next performed using ACK lysing buffer (Thermo Fisher Scientific, Waltham, MA, USA) followed by immune cell depletion using anti‐CD45 magnetic microbeads (Miltenyi Biotec, Bergisch Gladbach, Germany) in combination with LS columns (Miltenyi Biotec). Cells were resuspended in PBS with 0.04% non‐acetylated BSA before the single‐cell sequencing step using Chromium Single‐cell 3′ GEM, Library & Gel Bead Kit v3 (10× Genomics, Pleasanton, CA, USA) on a 10× Genomics Chromium Controller following the manufacturer's protocol. Single‐cell library preparation was performed using Chromium Single Cell 3′ GEM, Library & Gel Bead Kit v3 (10× Genomics; catalog no. 1000075) following the manufacturer's protocol. Sequencing was performed on an Illumina NextSeq 500. The transcriptome data was computationally analyzed to determine cell‐specific gene expression and cell identity as described before.^(^ ^24^ ^)^ Cell Ranger Single‐Cell Software Suite (10× Genomics) was used to perform sample demultiplexing, barcode processing, and single‐cell gene counting. The gene count matrix from the Cell Ranger pipeline was further analyzed using Seurat R toolkit^(^ ^25^ ^)^ as described before^(^ ^24^ ^)^ and various cell populations and their transcriptome profiles were determined. Clusters expressing chondrocyte markers *Acan*, *Sox9*, and *Col2a1* were extracted and further analyzed to determine differences between control and T1DM articular cartilage. RNA data are available for download from NCBI under accession no. GSE198837.

### 2.6 Immunofluorescent staining

Six‐micrometer sagittal sections from control and T1DM injured and uninjured mice were used for immunohistochemistry (IHC) as previously described.^(^ ^24^ ^)^ For antigen retrieval method, samples were treated with unitrieve for 30 minutes at 65°C. Primary antibodies MMP11 (MA5‐32285 [1:250]), MMP28 (18237‐1‐AP [1:50]), and Coll2 (MA5‐12789 [1:00]) (Thermo Fisher Scientific): CYTL1 (15856‐1‐AP [1:75]) (Proteintech, Rosemont, IL, USA) and MMP3 (Ab52915 [1:50]) (Abcam, Cambridge, UK) were incubated overnight at room temperature in a dark, humid chamber. Sample slides were then incubated at room temperature for 2 hours with the secondary antibodies (1:1000). Negative control slides were incubated with secondary antibody‐only. Stained slides were mounted with Prolong Gold with DAPI (Molecular Probes, Eugene, OR, USA). Slides were imaged using a Leica DM5000 microscope. ImagePro Plus V7.0 Software and a QIClick CCD camera (Qimaging, Surrey, Canada) were used for imaging and photo editing.

## 3 Results

### 3.1 STZ‐induced T1DM promotes cartilage degeneration in the knee joints of 16‐week‐old male mice

**Fig. 1:**
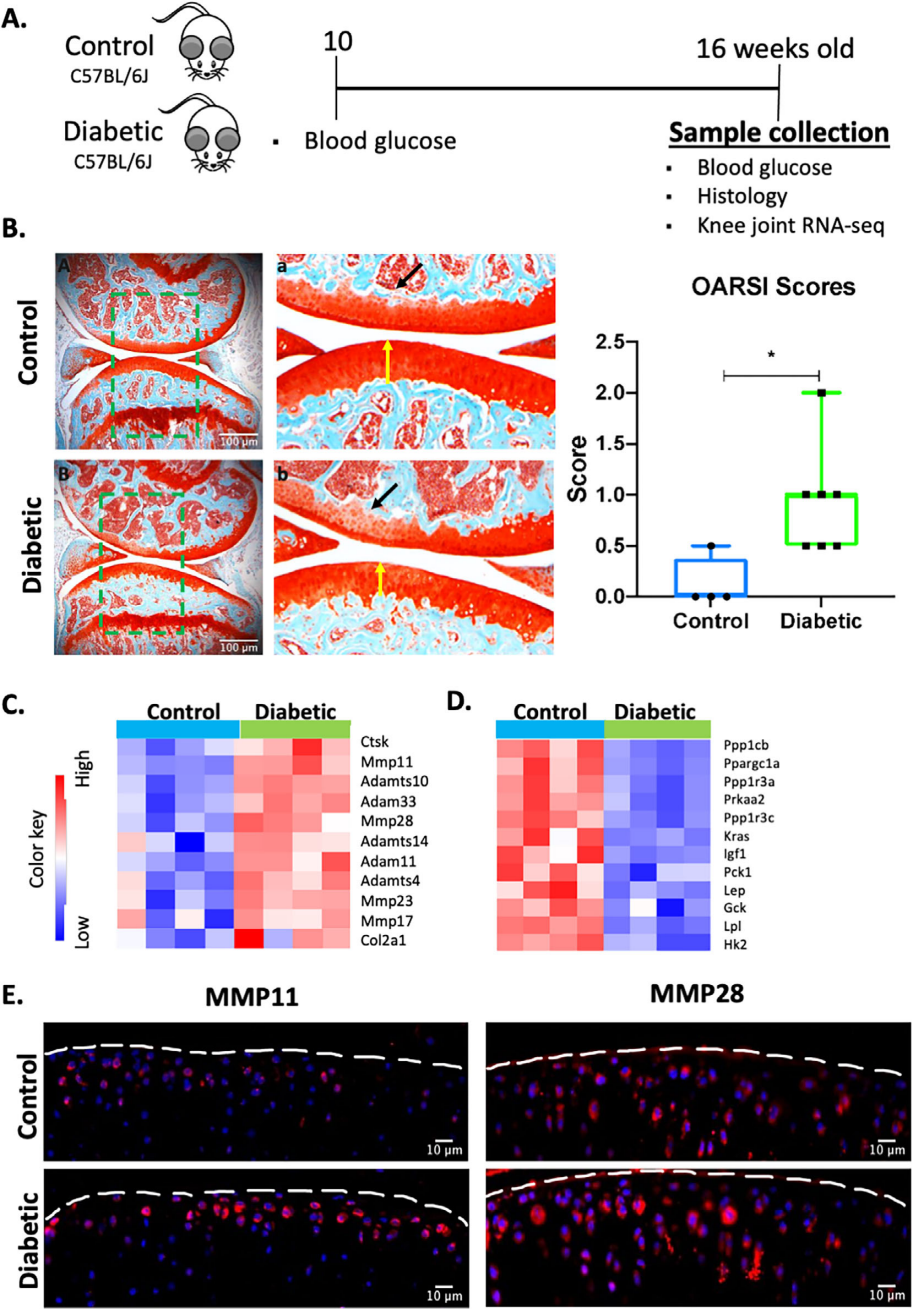
Type 1 diabetes mellitus promotes mild osteoarthritis in C57BL/6J male mice. (*A*) Schematic representation of the experimental design. (*B*) Histological evaluation of the knee joint of 16‐week‐old control and T1DM mice using Safranin O (red = cartilage) and Fast Green (green = surrounding tissue) (5× magnification; scale bars = 100 um). High‐magnification images corresponding to green boxes (A, B) are provided (a, b). Yellow arrows show cartilage thickness, while black arrows show lighter cartilage. OA severity was quantified using the Osteoarthritis Research Society International (OARSI) scoring system. (*C*) Heat map of selected matrix‐degrading enzymes and OA genes of interest. (*D*) Heat map of genes associated with insulin signaling and lipid metabolism. (*E*) Protein expression of MMP11 and MMP28. Blue = DAPI staining showing the nucleus; red = staining showing the protein of interest (20× magnification; scale bar = 10 μm). Bar graphs values are average ± standard error; control *n* = 5, diabetic *n* = 10 per group. Statistical analysis performed by *t* test. *p < 0.05.

To determine if T1DM is a risk factor for primary OA, we examined the knee joints of control and T1DM mice at 16 weeks of age (Fig. 1*A*). Non‐fasting blood glucose levels were significantly elevated (*p* < 0.005) at 10 and 16 weeks of age in the T1DM group, whereas control mice maintained lower blood glucose levels <300 mg/dL (Supplemental Fig. S1). Body weight was also assessed throughout the experiment, and consistent with previous reports(2 ^)^ STZ‐treated mice had lower body weight and maintained similar levels throughout the experiment when compared with the control mice (*p* < 0.0001; Supplemental Fig. S1). OA development was evaluated using Safranin O and Fast Green staining. Control mice exhibited healthy cartilage characterized by a smooth and strong Safranin O staining, and no bone degradation was visible on either the femoral condyle or in the tibia (Fig. 1*B*). In contrast, T1DM mice exhibited loss of Safranin O staining and minor fibrillation, suggesting the loss of proteoglycan content (Fig. 1*B*). The articular cartilage layer also appeared slightly thinner in some regions in the T1DM joints than in the controls (Fig. 1*B*, yellow arrows). OA severity was quantified using a modified Osteoarthritis Research Society International (OARSI) grading scale, and the OA score was found to be significantly higher (*p* < 0.05) in the T1DM group (Fig. 1*B*). These results confirm that T1DM is a risk factor for primary OA, and STZ‐induced T1DM mice display mild OA phenotypes at 16 weeks of age.

To understand how T1DM promotes primary OA, at the molecular level, we compared whole knee joint RNA‐sequences (RNA‐Seq) between 16‐week‐old control and T1DM mice. Computational analyses identified 951 up‐ and 1002 downregulated genes differentially expressed in T1DM joints relative to control joints (Supplemental Tables S1–S2). Among the upregulated genes, we identified several transcripts encoding for matrix‐degrading enzymes, including matrix metallopeptidase 11 (*Mmp11*), *Mmp23* and *Mmp28*, metalloproteinase 33 (*Adam 33*), metallopeptidase 14 (*Adamts 14*), *Adamts4*, and *Adamts10* (Fig. 1*C*). Cartilage‐specific genes such as collagen type II alpha 1 chain (*Col2a1*) were also affected by T1DM (Fig. 1*C*). Consistent with this gene expression data, immunohistochemical (IHC) staining confirmed higher protein levels of MMP11 and MMP28 in the articular cartilage of T1DM mice compared with controls (Fig. 1*E*), suggesting that these enzymes may be responsible for the emerging OA phenotype observed in the T1DM group. Other significantly upregulated genes of interest included extracellular matrix and skeletal development–associated genes (Supplemental Fig. S2). We and others have also previously shown that STZ‐induced T1DM decreases bone mineral density in mice;^2^, ^26^, ^27^^)^ in support of this phenotype, we also found elevated expression levels of several bone remodeling markers, including *Bglap*, *Acp5*, *Cstk*, *Oscar*, and *Pth1r*, in the T1DM group (Supplemental Fig. S2). Significantly downregulated genes were enriched in several functional categories, including markers of bone mineral content and skeletal muscle tissue development and morphology (Supplemental Fig. S2). We also observed downregulation of several genes associated with insulin signaling such as *Gck*, *Pck1*, *Igf*, and *Ppargc1a*, as well as *Lpl*, a regulator of lipid metabolism and leptin (*Lep*) (Fig. 1*D*).^(^ ^28^, ^29^, ^30^, ^31^, ^32^ ^)^

An ontology analysis identified type I interferon signaling pathway (GO:0060337), extracellular matrix organization (GO:0030198), response to cytokine (GO:0034097), and osteoclast differentiation (GO:0030316) as some of the key biological processes associated with upregulated genes while response to insulin (GO:0032868), regulation of lipid biosynthetic process (GO:0046890), and response to leptin (GO:0044321) were identified as enriched biological processes associated with downregulated genes (Supplemental Tables S3–S4).

### 3.2 Injury‐induced knee joint degeneration is blunted in T1DM mice

**Fig. 2:**
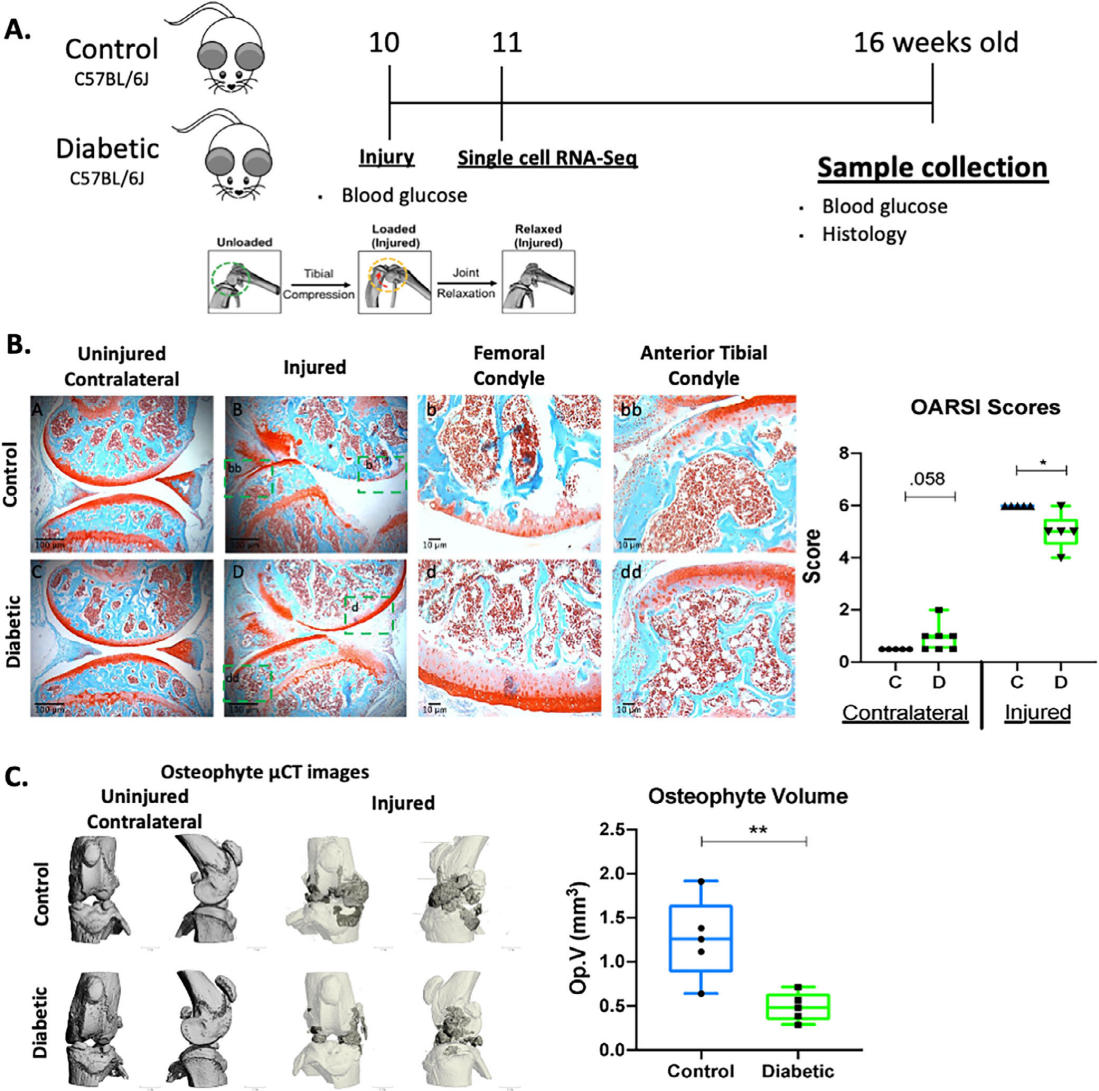
Preexisting type 1 diabetes mellitus prevents posttraumatic osteoarthritis in C57BL/6J male mice. (*A*) Schematic representation of the experimental design. (*B*) Histological evaluation of uninjured and injured control and diabetic mice at 6 weeks post‐injury using Safranin O (red = cartilage) and Fast Green (green = surrounding tissue) (5× and 20× magnification; scale bars = 100 and 10 μm). PTOA severity was quantified using the Osteoarthritis Research Society International (OARSI) scoring system. (*C*) Osteophyte imaging using μCT and osteophyte volume at 6 weeks post‐injury. Bar graph values are average ± standard error; *n* = 5 per group. Statistical analysis performed by *t* test. *p < 0.05; **p < 0.001.

Using a noninvasive tibial compression (TC) injury model in mice previously shown to reproducibly cause PTOA by 6 weeks post‐injury^(^ ^17^, ^33^ ^)^ we injured 10‐week‐old T1DM and C57BL/6J control mice, as previously described^(^ ^17^, ^18^, ^33^ ^)^ (Fig. 2*A*). Six weeks post‐injury, joints were harvested and PTOA phenotypes were examined by histology and μCT. Consistent with previous results,^(^ ^17^, ^18^, ^33^ ^)^ injured control mice displayed severe cartilage degradation with significant loss of Safranin O staining, and most of the medial femoral condyle was lacking the articular cartilage layer (Fig. 2*B*). In sharp contrast, injured T1DM mice more closely resembled the uninjured T1DM controls. Cartilage thickness was preserved, and while some proteoglycan staining loss was observed in the deep layer, the superficial layer was unchanged, suggesting a significantly slower progression to PTOA in the T1DM mice (Fig. 2*B*). Quantification of OA severity using the OARSI grading scale system showed significantly higher cartilage score in the injured control group than in the injured T1DM group (Fig. 2*B*; *p* < 0.05). Subchondral trabecular bone mass and osteophyte volume was also quantified by μCT. Consistent with prior reports that T1DM promotes bone loss,^(^ ^2^, ^26^ ^)^ uninjured T1DM mice had significantly less BV/TV and less trabecular thickness compared with the control mice. Although joint injury also significantly affected bone mass in the control mice, trabecular bone mass was not affected in the T1DM group relative to the controls. No additional significant bone loss was observed in the subchondral bone of injured T1DM mice when compared with uninjured T1DM or injured control mice (Table 1). T1DM mice were also protected from osteophyte formation in response to injury, where significantly less ectopic bone was quantified around the injured joints of the T1DM group (Fig. 2*C*). These results suggest a slower progression of PTOA in T1DM mice.

**Table 1:** Femoral Subchondral Bone Parameters in Control and Diabetic Uninjured and Injured Mice

Femoral subchondral bone parameters				
	Uninjured		Injured	
	Control (*n* = 5)	Diabetic (*n* = 5)	Control (*n* = 5)	Diabetic (*n* = 5)
Parameter				
BV/TV %	37.79 ± 0.83	30.84 ± 2.11*	30.26 ± 0.64*	27.41 ± 1.35
Tb.Th (mm)	0.063 ± 0.001	0.054 ± 0.003*	0.058 ± 0.001*	0.049 ± 0.002
Tn.N (1/mm)	5.97 ± 0.06	5.75 ± 0.10	5.45 ± 0.08*	5.63 ± 0.11
Tb.Sp (mm)	0.16 ± 0.002	0.16 ± 0.004	0.17 ± 0.002*	0.16 ± 0.003

BV/TV = trabecular bone volume per total volume; Tb.Th = trabecular thickness; Tb.N = trabecular number; Tb.Sp = trabecular spacing.

* *p* < 0.05 compared with uninjured control.

### 3.3 scRNA‐Seq analysis identified more chondrocyte in the T1DM mouse knee joints

**Fig. 3:**
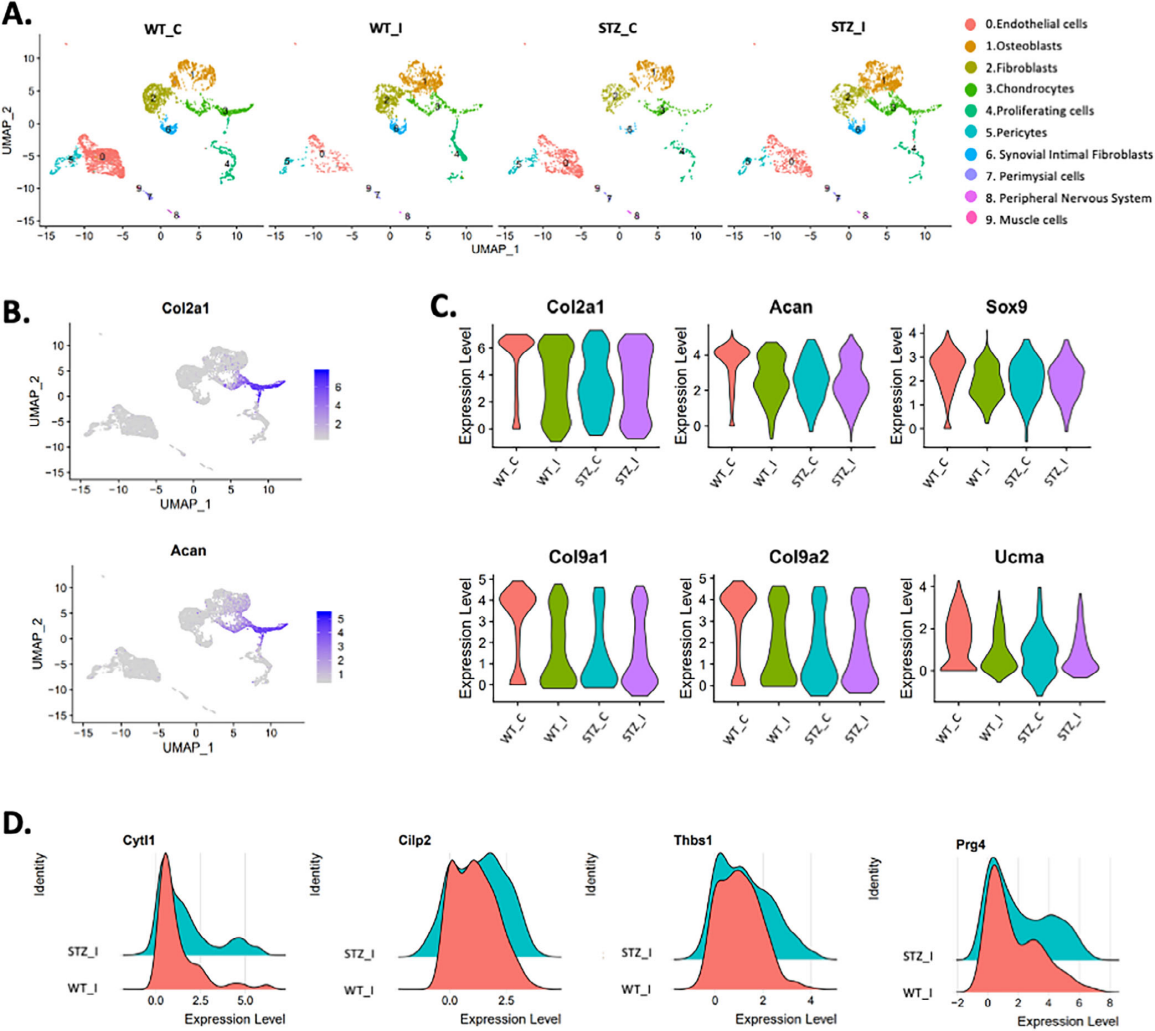
Single‐cell RNA‐Seq reveals more chondrocytes in the type 1 diabetic mellitus injured mice. (*A*) Cell clusters from scRNA‐Seq analysis visualized by uniform manifold approximation and projection (UMAP) plot. Colors indicate clusters of various cell types. (*B*) Feature plots showing the expression of the chondrocyte markers *Col2a1* and *Acan*; high expression (blue), low expression (gray). (*C*) Violin plot showing the expression of *Col2a1*, *Acan*, *Sox9*, *Col9a1*, *Col9a2*, and *Ucma*. (*D*) Ridge plots showing the expression of several genes between C57BL/6J control and STZ injured mice.

To determine the mechanism by which T1DM prevents cartilage degradation after injury, we performed single‐cell RNA sequencing (scRNA‐Seq) analysis of the knee joint at 7 days post‐injury. ScRNA‐Seq was conducted in viable, immune, and blood depleted cells isolated from uninjured and injured control and T1DM mice. A graph‐based clustering of the stroma cells using Seurat resulted in 9 cell clusters with distinct gene expression profiles (Figs. 3*A* and Supplemental S3). Cluster 0 showed high expression of the endothelial cell markers Pecam1 and VE‐Cadherin (*Cdh5*).^(^ ^34^ ^)^ Cluster 1 expressed high levels of well‐known osteoblast markers osteocalcin (*Bglap*) and *Col1a1* and was labeled osteoblast.^(^ ^35^ ^)^ Cluster 2 was labeled fibroblast based on enrichment of genes *Dcn* and *Clec3b*.^(^ ^34^ ^)^ Cluster 3 showed enrichment for *Acan* and *Col2a1* and was annotated chondrocytes.^(^ ^24^ ^)^ Cluster 4 showed high expression of proliferative markers *Mki67* and *Top2a* and was named proliferative cells.^(^ ^36^ ^)^ Cluster 5, named pericytes, showed high levels of *Myh11* and *Rgs5*.^(^ ^37^, ^38^ ^)^ Cluster 6 was named synovial intimal fibroblasts (SIFs) because of the expression of fibroblast markers lubricin (*Prg4*) and *Htra4*.^(^ ^39^ ^)^ Cluster 7 named perimysial cells expressed high levels of *Myod1* and *Chodl*.^(^ ^40^, ^41^ ^)^ Cluster 8 peripheral nervous system (PNS) cells showed high expression of *Mpz* and *Mbp*.^(^ ^42^ ^)^ Cluster 9 muscle cells expressed high levels of *Tnnc2* and *Myl1*.^(^ ^43^, ^44^ ^)^ We then extracted and reexamined in more detail cluster number 3, which expressed the chondrocyte markers *Acan* and *Col2a1* (Fig. 3 *A*, *B*).

**Fig. 4:**
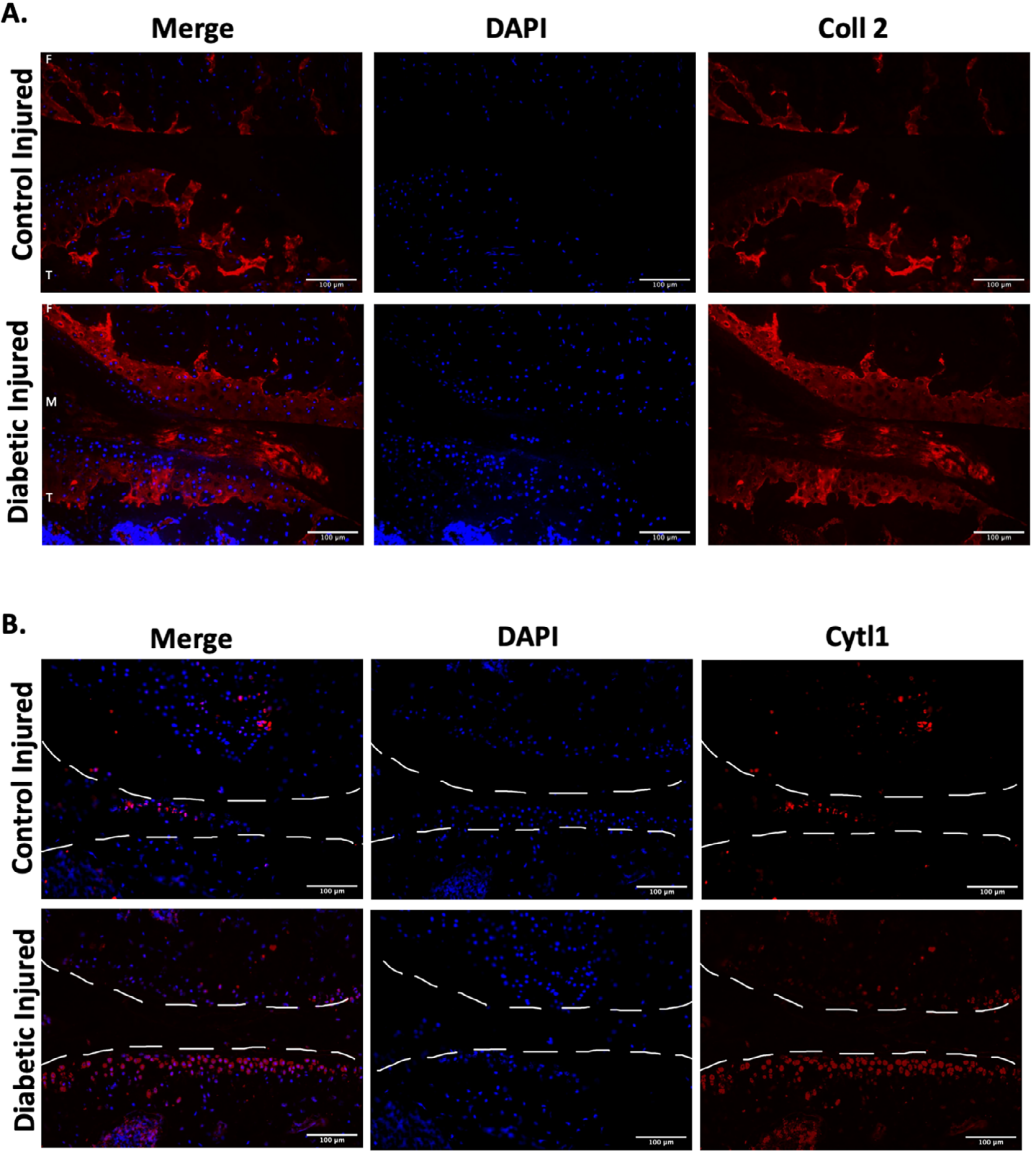
Type 1 diabetes injured mice express higher protein levels of chondrocyte markers. (*A*) Protein expression of collagen 2 (Coll 2). Blue = DAPI staining showing the nucleus; red = staining showing Coll 2 (20× magnification; scale bar = 100 μm). (*B*) Protein expression of cytokine like 1 (Cytl1). Blue = DAPI staining showing the nucleus; red = staining showing Cytl1 (20× magnification; scale bar = 100 μm).

After injury, there was a significant reduction in *Col2a1*, *Sox9*, and *Acan* expression in both T1DM and C57BL/6J control groups (Fig. 3*C*). However, we also observed that T1DM injured mice had higher levels of *Col2a1* and *Acan* when compared with the injured C57BL/6J control group (Fig. 3*C*). In uninjured healthy chondrocytes, we observed higher expression of cartilage‐associated genes *Col9a1*, *Col9a2*, and *Ucma* compared with other groups (Fig. 3*C*). We also observed an increase in frequency of cells expressing several genes dysregulated in OA, such as *Cilp2*, *Prg4*, and *Thbs1* in the STZ injured groups when compared with C57BL/6J injured mice (Fig. 3*D*). Interestingly, the T1DM injured group had more *Cytl*‐expressing cells (Fig. 3*D*). In prior work, we have shown high expression of *Cytl1* in the mid layer of the articular cartilage and that this expression was reduced as a result of injury and aging.^(^ ^24^, ^33^ ^)^ Consistent with lower cartilage degradation in the injured T1MD mice, this group also showed significantly higher levels of type II collagen (Col2) protein expression when compared with injured control and with uninjured T1D controls (Fig. 4*A*). Furthermore, IHCs confirmed that T1DM injured joints preserved a significantly higher number of Cytl1‐positive cells (Fig. 4*B*). An increased number of *Col2a1‐*, *Acan‐*, and *Cytl1*‐expressing chondrocytes in the T1DM group therefore indicates slower OA progression in T1DM group.

## 4 Discussion

Diabetes and OA are two conditions that can significantly impact a patient's quality of life. As the life expectancy of the elderly population increases, the coexistence of OA and diabetes becomes more prevalent. In addition, >70% of people over the age of 65 have evidence of radiological knee OA. During the last decade, several studies have investigated the relationship between diabetes and OA; however, the results have been inconsistent and even contradictory. In the present study, we show, unexpectedly, that systemic preexisting T1DM while slightly elevating the risk for primary OA development also confers a protective effect against trauma‐induced osteoarthritis. T1DM slows the joint degeneration initiated by anterior cruciate ligament (ACL) rupture, slowing down cartilage degeneration, subchondral bone loss, and osteophyte formation.

Several studies in humans have addressed whether diabetes can be considered a risk factor for OA. However, studies in humans are complex and care should be taken when interpreting the results. In addition, OA and diabetes share several additional common risk factors, such as obesity and aging, which may further skew data analysis and the interpretation of the results.^(^ ^45^ ^)^ Our histological analysis in 16‐week‐old male mice showed a mild OA phenotype in the T1DM diabetic group when compared with the control group, suggesting an effect of T1DM on spontaneous (idiopathic). Our findings corroborate several studies in mice in which loss of chondrocytes and cartilage tissue in the joints of diabetic animals were reported.^(^ ^14^, ^15^, ^46^, ^47^ ^)^ However, the severity of OA in our study was much less preeminent than in other studies. This difference in OA severity and responses in the T1DM animals may be explained by several factors such as the use of different animal strains, the age of the animal, the duration of the experiment, type of diabetic model used, and the type of histological assessment of OA. One of the strengths of our study is that we used the STZ‐induced diabetic animal model, which does not promote a gain in body weight, completely separating the effect of body weight mass versus the effect of T1DM on OA. An additional strength of our study is the use of a noninvasive injury model, which eliminates the variability and robust inflammatory response produced by invasive surgical methods used to sever ligaments in the joint.

By bulk RNA‐Seq, we identified several OA‐related genes such as *Mmp11*, *Mmp23*, *Mmp28*, *Adam33*, *Adamts4*, and *Adamts14* that may be responsible for the emerging OA phenotype observed in the T1DM group. However, one of the limitations of doing bulk RNA‐Seq is the inability to tease out the contribution of individual cell type/subtype‐specific gene expressions changes. Therefore, it is possible that our bulk gene expression data are not only from the articular tissue but include some cells from adjacent tissues such as the subchondral bone, bone marrow, muscle, and synovium. To overcome this challenge, we examined tissue‐specific expression of genes using single‐cell RNA‐Seq for the second part of this project.

Despite findings that T1DM promotes mild spontaneous OA, our data also unexpectantly showed that preexisting T1DM blunts the development of PTOA after noninvasive ACL rupture. Although we identified a significant reduction in *Col2a1*, *Sox9*, and *Acan* expression in both T1DM and C57BL/6J control injured groups, the T1DM injured joints displayed a significant increase in the number of *Col2a1‐*, *Sox9‐*, and *Acan 1*‐expressing chondrocytes when compared with the injured control joints at 7 days post‐injury. We chose to perform our scRNA‐Seq analysis at 7 days post‐injury because previous data from our lab have determined the most differentially expressed genes occur at this specific time point.^(^ ^48^ ^)^ *Col2a1*, *Sox9*, and *Acan* are all well‐known key regulators of cartilage development and homeostasis.^(^ ^49^, ^50^, ^51^ ^)^ In addition, our lab has also shown a reduction in the expression of these genes in aging mice that have PTOA.^(^ ^33^ ^)^ *Cytl1*, a gene that has recently been shown to be involved in chondrocyte and cartilage development, was also highly expressed in the T1DM injured mice.^(^ ^24^, ^33^, ^52^, ^53^ ^)^ *Cytl1* expression is also downregulated in injured and 62‐week‐old mice^(^ ^33^ ^)^ and *Cytl1* knock‐out mice are more sensitive to OA than wild‐type mice.^(^ ^52^ ^)^ Consistent with our single‐cell RNA‐Seq data, immunohistochemical analysis showed that Cytl1 expression is blunted in the T1DM injured group and reduced in the injured control group. Elevated expression of *Cytl1‐*expressing chondrocytes in the T1DM injured mice suggest a potential role of these cells in the prevention of PTOA development by T1DM. The injured T1DM mice also exhibited less osteophyte formation than the C57BL/6J control injured group, and there is a possibility that osteophyte formation positively correlates with disease severity, and hence there are some synergistic effects. However, in prior work, we did find exceptions to this observation; for example, STR/ort strain of mice, which are genetically susceptible to OA and develop spontaneous OA, upon injury, developed less osteophytes than injured C57BL/6J control injured groups.^(^ ^33^ ^)^ Similarly, C57BL/6J injured joints from mice treated with LPS, while exhibiting a more severe PTOA phenotype than the saline‐treated C57BL/6J injured group, also had less osteophyte formation than the group without LPS.^(^ ^54^ ^)^ These examples suggest that cartilage‐osteophyte interactions are more complex and may involve several cell types and multiple modeling and remodeling steps to achieve the observable phenotypes.

When animals were euthanized, we also observed less subcutaneous white adipose tissue (WTA) and our bulk RNA‐Seq also show lower expression of *Lpl*, a regulator of lipid metabolism and leptin (*Lep*) in the T1DM uninjured mice. Our results are consistent with prior reports where rats treated with STZ showed a decrease in parametrial (PWAT) and retroperitoneal (RWAT) white adipose tissues.^(^ ^55^ ^)^ A different study also reported that T1DM‐induced rats showed marked reductions in fat mass and mean of fat cell diameter at all subcutaneous (SC), proximal epididymal (PE), distal epididymal (DE), perirenal (PR), and retroperitoneal (RP) fat depots.^(^ ^56^ ^)^ Parting from the findings that less adipose tissue can prevent OA^(^ ^57^ ^)^ we would have expected less OA in the T1DM animals; however, our data show more OA in T1DM mice, suggesting no correlation between fat and OA development in our model.

In conclusion, our study is the first to describe molecular changes at single‐cell resolution in the knee joint of T1DM animals in context of OA and PTOA. We also investigated gene expression at the single‐cell level, and we have shown the individual cell type/subtype that might be involved in slowing PTOA progression in the T1DM joint environment. Although we found that T1DM promotes mild OA and prevents PTOA, it remains to be elucidated whether T2DM can also have similar effects. Human studies looking at the role of DM on OA have yielded inconsistent and conflicting results with some showing a significant role for DM in promoting OA, while others finding no significant interactions between these two conditions. Interestingly, in our study, we found that T1DM can promote primary OA, while preventing or slowing down secondary OA, suggesting that different mechanisms are at play by which primary and secondary OA develop. Furthermore, to our knowledge, no study to date has addressed the effects of T1DM on PTOA in humans; this is a knowledge gap that we hope will be examined by future clinical work. Our study highlights the importance of studying T1DM in the context of both OA and PTOA and suggests that while T1DM may increase susceptibility to degenerative OA, upon injury, the PTOA disease progression is blunted in the diabetic environment.

## Disclosures

All authors state that they have no conflicts of interest.

## Author Contributions

**Naiomy D Rios‐Arce:** Conceptualization; data curation; formal analysis; investigation; methodology; validation; visualization; writing – original draft. **Deepa K Murugesh:** Data curation; investigation; methodology; writing – review and editing. **Nicholas R Hum:** Data curation; investigation; writing – review and editing. **Aimy Sebastian:** Data curation; formal analysis; visualization; writing – review and editing. **Elias H Jbeily:** Data curation. **Blaine A Christiansen:** Data curation; funding acquisition; project administration; resources; writing – review and editing. **Gabriela G Loots:** Conceptualization; formal analysis; funding acquisition; methodology; project administration; resources; supervision; visualization; writing – original draft.

5

### Peer Review

The peer review history for this article is available at https://publons.com/publon/10.1002/jbm4.10625.

## Supporting information


**Appendix S1**. Supplemental Information

## References

[1] Xu G , Liu B , Sun Y , et al. Prevalence of diagnosed type 1 and type 2 diabetes among US adults in 2016 and 2017: population based study. BMJ. 2018;362:k1497. 30181166 10.1136/bmj.k1497 PMC6122253

[2] Rios‐Arce ND , Dagenais A , Feenstra D , et al. Loss of interleukin‐10 exacerbates early Type‐1 diabetes‐induced bone loss. J Cell Physiol. 2020;235(3):2350‐2365. 31538345 10.1002/jcp.29141 PMC6899206

[3] Louati K , Vidal C , Berenbaum F , Sellam J . Association between diabetes mellitus and osteoarthritis: systematic literature review and meta‐analysis. RMD Open. 2015;1(1):e000077. 26535137 10.1136/rmdopen-2015-000077 PMC4613158

[4] Williams MF , London DA , Husni EM , Navaneethan S , Kashyap SR . Type 2 diabetes and osteoarthritis: a systematic review and meta‐analysis. J Diabetes Complications. 2016;30(5):944‐950. 27114387 10.1016/j.jdiacomp.2016.02.016

[5] Leung YY , Allen JC , Ang LW , Yuan JM , Koh WP . Diabetes mellitus and the risk of total knee replacement among Chinese in Singapore, the Singapore Chinese Health Study. Sci Rep. 2017;7:40671. 28084472 10.1038/srep40671 PMC5233971

[6] Nielen JTH , Emans PJ , van den Bemt B , et al. Association of type 2 diabetes mellitus with self‐reported knee pain and clinical knee osteoarthritis: the Maastricht study. Diabetes Metab. 2018;44(3):296‐299. 29422359 10.1016/j.diabet.2018.01.013

[7] Dawson LP , Fairley JL , Papandony MC , Hussain SM , Cicuttini FM , Wluka AE . Is abnormal glucose tolerance or diabetes a risk factor for knee, hip, or hand osteoarthritis? A systematic review. Semin Arthritis Rheum. 2018;48(2):176‐189. 29550110 10.1016/j.semarthrit.2018.02.008

[8] Zhang Y , Jordan JM . Epidemiology of osteoarthritis. Clin Geriatr Med. 2010;26(3):355‐369. 20699159 10.1016/j.cger.2010.03.001 PMC2920533

[9] Chen D , Shen J , Zhao W , et al. Osteoarthritis: toward a comprehensive understanding of pathological mechanism. Bone Res. 2017;5:16044. 28149655 10.1038/boneres.2016.44 PMC5240031

[10] Lohmander LS , Englund PM , Dahl LL , Roos EM . The long‐term consequence of anterior cruciate ligament and meniscus injuries: osteoarthritis. Am J Sports Med. 2007;35(10):1756‐1769. 17761605 10.1177/0363546507307396

[11] Khor A , Ma CA , Hong C , Hui LL , Leung YY . Diabetes mellitus is not a risk factor for osteoarthritis. RMD Open. 2020;6(1):e001030. 32060073 10.1136/rmdopen-2019-001030 PMC7046958

[12] Neumann J , Hofmann FC , Heilmeier U , et al. Type 2 diabetes patients have accelerated cartilage matrix degeneration compared to diabetes free controls: data from the osteoarthritis initiative. Osteoarthr Cartil. 2018;26(6):751‐761. 10.1016/j.joca.2018.03.010 PMC5962437 29605381

[13] Telzrow RW , Snyder DM , Tronick E , Als H , Brazelton TB . The behavior of jaundiced infants undergoing phototherapy. Dev Med Child Neurol. 1980;22(3):317‐326. 7390029 10.1111/j.1469-8749.1980.tb03711.x

[14] Chen YJ , Chan DC , Lan KC , et al. PPARgamma is involved in the hyperglycemia‐induced inflammatory responses and collagen degradation in human chondrocytes and diabetic mouse cartilages. J Orthop Res. 2015;33(3):373‐381. 25410618 10.1002/jor.22770

[15] Dubey NK , Wei HJ , Yu SH , et al. Adipose‐derived stem cells attenuates diabetic osteoarthritis via inhibition of glycation‐mediated inflammatory Cascade. Aging Dis. 2019;10(3):483‐496. 31164994 10.14336/AD.2018.0616 PMC6538220

[16] Graham ML , Janecek JL , Kittredge JA , Hering BJ , Schuurman HJ . The streptozotocin‐induced diabetic nude mouse model: differences between animals from different sources. Comp Med. 2011;61(4):356‐360. 22330251 PMC3155402

[17] Chang JC , Sebastian A , Murugesh DK , et al. Global molecular changes in a tibial compression induced ACL rupture model of post‐traumatic osteoarthritis. J Orthop Res. 2017;35(3):474‐485. 27088242 10.1002/jor.23263 PMC5363336

[18] Christiansen BA , Guilak F , Lockwood KA , et al. Non‐invasive mouse models of post‐traumatic osteoarthritis. Osteoarthr Cartil. 2015;23(10):1627‐1638. 10.1016/j.joca.2015.05.009 PMC4577460 26003950

[19] Goel PN , Egol AJ , Moharrer Y , Brandfield‐Harvey B , Ahn J , Ashley JW . Notch signaling inhibition protects against LPS mediated osteolysis. Biochem Biophys Res Commun. 2019;515(4):538‐543. 31176486 10.1016/j.bbrc.2019.05.166 PMC7228139

[20] Glasson SS , Chambers MG , Van Den Berg WB , Little CB . The OARSI histopathology initiative—recommendations for histological assessments of osteoarthritis in the mouse. Osteoarthr Cartil. 2010;18(Suppl 3):S17‐S23. 10.1016/j.joca.2010.05.025 20864019

[21] Dobin A , Davis CA , Schlesinger F , et al. STAR: ultrafast universal RNA‐seq aligner. Bioinformatics. 2013;29(1):15‐21. 23104886 10.1093/bioinformatics/bts635 PMC3530905

[22] Robinson MD , McCarthy DJ , Smyth GK . edgeR: a bioconductor package for differential expression analysis of digital gene expression data. Bioinformatics. 2010;26(1):139‐140. 19910308 10.1093/bioinformatics/btp616 PMC2796818

[23] Kuleshov MV , Jones MR , Rouillard AD , et al. Enrichr: a comprehensive gene set enrichment analysis web server 2016 update. Nucl Acids Res. 2016;44(W1):W90‐W97. 27141961 10.1093/nar/gkw377 PMC4987924

[24] Sebastian A , McCool JL , Hum NR , et al. Single‐cell RNA‐Seq reveals transcriptomic heterogeneity and post‐traumatic osteoarthritis‐associated early molecular changes in mouse articular chondrocytes. Cells. 2021;10(6):1462. 34200880 10.3390/cells10061462 PMC8230441

[25] Stuart T , Butler A , Hoffman P , et al. Comprehensive integration of single‐cell data. Cell. 2019;177(7):1888‐1902.e21. 31178118 10.1016/j.cell.2019.05.031 PMC6687398

[26] Motyl K , McCabe LR . Streptozotocin, type I diabetes severity and bone. Biol Proced Online. 2009;11:296‐315. 19495918 10.1007/s12575-009-9000-5 PMC3055251

[27] Yee CS , Xie L , Hatsell S , et al. Sclerostin antibody treatment improves fracture outcomes in a type I diabetic mouse model. Bone. 2016;82:122‐134. 25952969 10.1016/j.bone.2015.04.048 PMC4635060

[28] Matschinsky FM , Wilson DF . The central role of glucokinase in glucose homeostasis: a perspective 50 years after demonstrating the presence of the enzyme in islets of Langerhans. Front Physiol. 2019;10:148. 30949058 10.3389/fphys.2019.00148 PMC6435959

[29] Millward CA , Desantis D , Hsieh CW , et al. Phosphoenolpyruvate carboxykinase (Pck1) helps regulate the triglyceride/fatty acid cycle and development of insulin resistance in mice. J Lipid Res. 2010;51(6):1452‐1463. 20124556 10.1194/jlr.M005363 PMC3035508

[30] Zielinski R , Przytycki PF , Zheng J , Zhang D , Przytycka TM , Capala J . The crosstalk between EGF, IGF, and insulin cell signaling pathways—computational and experimental analysis. BMC Syst Biol. 2009;3:88. 19732446 10.1186/1752-0509-3-88 PMC2751744

[31] Santos JL , Krause BJ , Cataldo LR , et al. PPARGC1A gene promoter methylation as a biomarker of insulin secretion and sensitivity in response to glucose challenges. Nutrients. 2020;12(9):2790. 10.3390/nu12092790 PMC7551463 32933059

[32] Hynes GR , Jones PJ . Leptin and its role in lipid metabolism. Curr Opin Lipidol. 2001;12(3):321‐327. 11353336 10.1097/00041433-200106000-00012

[33] Sebastian A , Murugesh DK , Mendez ME , et al. Global gene expression analysis identifies age‐related differences in knee joint transcriptome during the development of post‐traumatic osteoarthritis in mice. Int J Mol Sci. 2020;21(1):364. 10.3390/ijms21010364 PMC6982134 31935848

[34] Sebastian A , Hum NR , Martin KA , et al. Single‐cell transcriptomic analysis of tumor‐derived fibroblasts and normal tissue‐resident fibroblasts reveals fibroblast heterogeneity in breast cancer. Cancers (Basel). 2020;12(5):1307. 10.3390/cancers12051307 PMC7281266 32455670

[35] Tikhonova AN , Dolgalev I , Hu H , et al. The bone marrow microenvironment at single‐cell resolution. Nature. 2019;569(7755):222‐228. 30971824 10.1038/s41586-019-1104-8 PMC6607432

[36] Scott RE , Ghule PN , Stein JL , Stein GS . Cell cycle gene expression networks discovered using systems biology: significance in carcinogenesis. J Cell Physiol. 2015;230(10):2533‐2542. 25808367 10.1002/jcp.24990 PMC4481160

[37] Esteves CL , Donadeu FX . Pericytes and their potential in regenerative medicine across species. Cytometry A. 2018;93(1):50‐59. 28941046 10.1002/cyto.a.23243

[38] Shen J , Shrestha S , Yen YH , et al. The pericyte antigen RGS5 in perivascular soft tissue tumors. Hum Pathol. 2016;47(1):121‐131. 26558691 10.1016/j.humpath.2015.09.013 PMC4861638

[39] Chou CH , Jain V , Gibson J , et al. Synovial cell cross‐talk with cartilage plays a major role in the pathogenesis of osteoarthritis. Sci Rep. 2020;10(1):10868. 32616761 10.1038/s41598-020-67730-y PMC7331607

[40] Han X , Feng J , Guo T , et al. Runx2‐Twist1 interaction coordinates cranial neural crest guidance of soft palate myogenesis. Elife. 2021;10:e62387. 33482080 10.7554/eLife.62387 PMC7826157

[41] Muhl L , Genove G , Leptidis S , et al. Single‐cell analysis uncovers fibroblast heterogeneity and criteria for fibroblast and mural cell identification and discrimination. Nat Commun. 2020;11(1):3953. 32769974 10.1038/s41467-020-17740-1 PMC7414220

[42] Wolbert J , Li X , Heming M , et al. Redefining the heterogeneity of peripheral nerve cells in health and autoimmunity. Proc Natl Acad Sci U S A. 2020;117(17):9466‐9476. 32295886 10.1073/pnas.1912139117 PMC7196786

[43] Li Y , Chen Y , Li J , et al. Molecular characterization, expression profile and polymorphisms of the porcine TNNC2 gene. Hereditas. 2008;145(6):274‐282. 19200139 10.1111/j.1601-5223.2008.02083.x

[44] Ling F , Fang W , Chen Y , et al. Identification of novel transcripts from the porcine MYL1 gene and initial characterization of its promoters. Mol Cell Biochem. 2010;343(1–2):239‐247. 20563743 10.1007/s11010-010-0519-1

[45] Leung YY , Allen JC Jr , Noviani M , et al. Association between body mass index and risk of total knee replacement, the Singapore Chinese Health Study. Osteoarthr Cartil. 2015;23(1):41‐47. 10.1016/j.joca.2014.10.011 PMC4275403 25450848

[46] Chan DC , Chiu CY , Lan KC , Weng TI , Yang RS , Liu SH . Transplantation of human skeletal muscle‐derived progenitor cells ameliorates knee osteoarthritis in streptozotocin‐induced diabetic mice. J Orthop Res. 2017;35(9):1886‐1893. 27935109 10.1002/jor.23503

[47] Ribeiro M , Lopez de Figueroa P , Nogueira‐Recalde U , et al. Diabetes‐accelerated experimental osteoarthritis is prevented by autophagy activation. Osteoarthr Cartil. 2016;24(12):2116‐2125. 10.1016/j.joca.2016.06.019 27390029

[48] Sebastian A , Chang JC , Mendez ME , et al. Comparative transcriptomics identifies novel genes and pathways involved in post‐traumatic osteoarthritis development and progression. Int J Mol Sci. 2018;19(9):2657. 10.3390/ijms19092657 PMC6163882 30205482

[49] Bi W , Deng JM , Zhang Z , Behringer RR , de Crombrugghe B . Sox9 is required for cartilage formation. Nat Genet. 1999;22(1):85‐89. 10319868 10.1038/8792

[50] Fassler R , Schnegelsberg PN , Dausman J , et al. Mice lacking alpha 1 (IX) collagen develop noninflammatory degenerative joint disease. Proc Natl Acad Sci U S A. 1994;91(11):5070‐5074. 8197187 10.1073/pnas.91.11.5070 PMC43933

[51] Arikawa‐Hirasawa E , Watanabe H , Takami H , Hassell JR , Yamada Y . Perlecan is essential for cartilage and cephalic development. Nat Genet. 1999;23(3):354‐358. 10545953 10.1038/15537

[52] Kim JS , Ryoo ZY , Chun JS . Cytokine‐like 1 (Cytl1) regulates the chondrogenesis of mesenchymal cells. J Biol Chem. 2007;282(40):29359‐29367. 17644814 10.1074/jbc.M700965200

[53] Jeon J , Oh H , Lee G , et al. Cytokine‐like 1 knock‐out mice (Cytl1−/−) show normal cartilage and bone development but exhibit augmented osteoarthritic cartilage destruction. J Biol Chem. 2011;286(31):27206‐27213. 21652695 10.1074/jbc.M111.218065 PMC3149314

[54] Mendez ME , Sebastian A , Murugesh DK , et al. LPS‐induced inflammation prior to injury exacerbates the development of post‐traumatic osteoarthritis in mice. J Bone Miner Res. 2020;35(11):2229‐2241. 32564401 10.1002/jbmr.4117 PMC7689775

[55] Geloen A , Roy PE , Bukowiecki LJ . Regression of white adipose tissue in diabetic rats. Am J Physiol. 1989;257(4 Pt 1):E547‐E553. 2801936 10.1152/ajpendo.1989.257.4.E547

[56] Ghorbani A , Varedi M , Hadjzadeh MA , Omrani GH . Type‐1 diabetes induces depot‐specific alterations in adipocyte diameter and mass of adipose tissues in the rat. Exp Clin Endocrinol Diabetes. 2010;118(7):442‐448. 20198560 10.1055/s-0030-1247566

[57] Collins KH , Lenz KL , Pollitt EN , et al. Adipose tissue is a critical regulator of osteoarthritis. Proc Natl Acad Sci U S A. 2021;118(1):e202109611. 10.1073/pnas.2021096118 PMC7817130 33443201

