# Supplementary material for: Preexisting Type 1 Diabetes Mellitus Blunts the Development of Posttraumatic Osteoarthritis
Source: JBMR Plus. 2022 Apr 19;6(5):e10625. doi: 10.1002/jbm4.10625 (PMC9059474; doi:10.1002/jbm4.10625)
Supplement: Supplementary file 1 — Appendix S1. Supplemental Information [file JBM4-6-e10625-s001.docx]

**Supplementary Figure 1.**


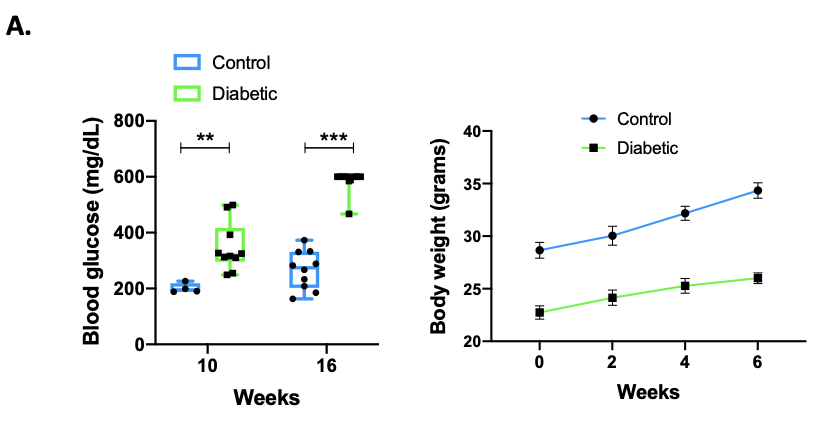


**Supp. Figure 1. Streptozotocin treatment effects on blood glucose and body weight.** (A) Blood glucose and body weight measurements through the experiment. Bar graphs values are average ± standard error; control n=5, diabetic n=10 per group. Statistical analysis performed by t-test. **p*<0.05, ***p*<0.001

**Supplementary Figure 2.**

**
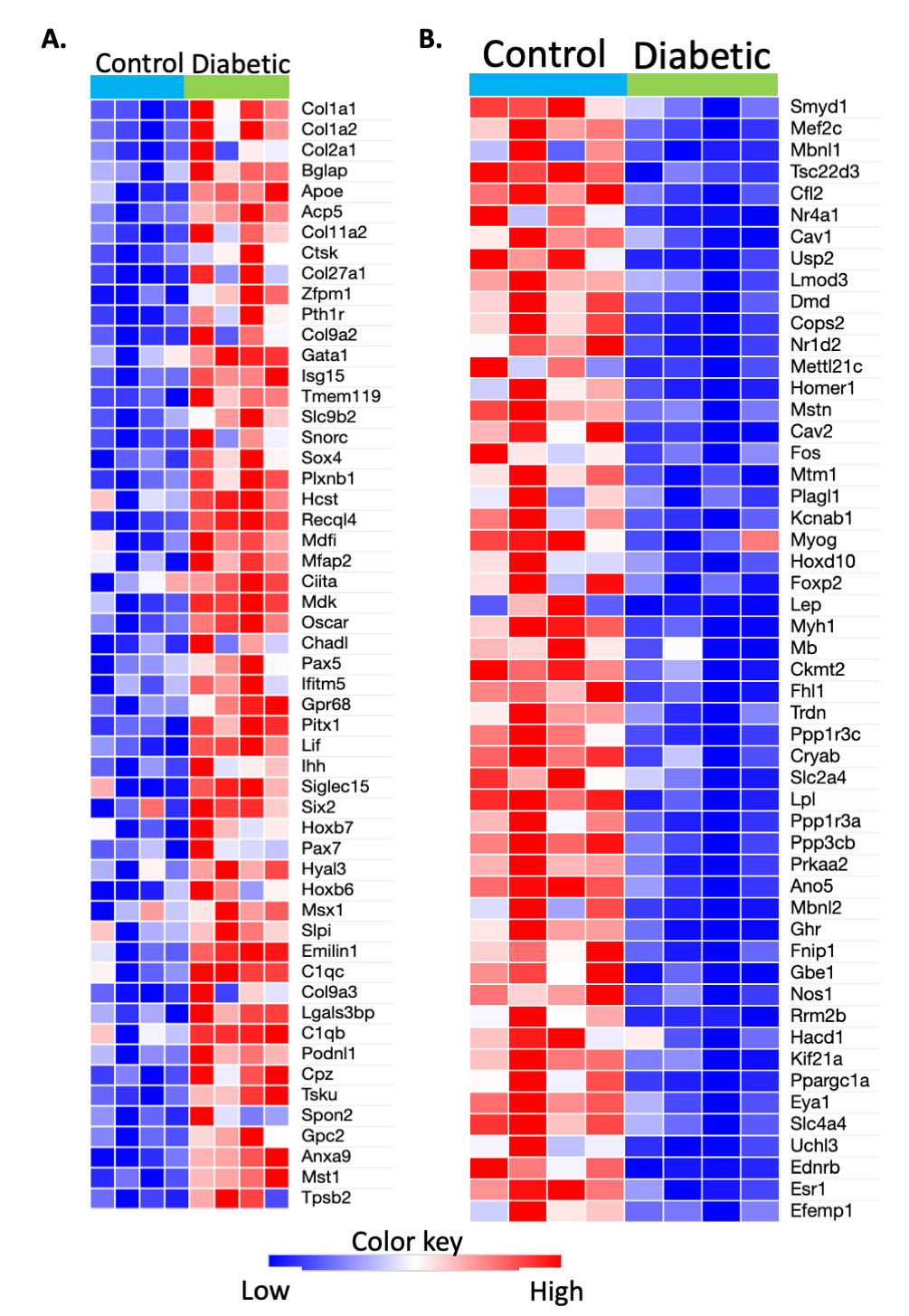
**

**Supp. Figure 2. T1D mice have elevated expression of bone remodeling markers.** (A) Heat map of selected bone remodeling markers. (B) Heat map of selected bone mineral content and skeletal muscle tissue development and morphology associated genes.

**Supplementary Figure 3.**

**
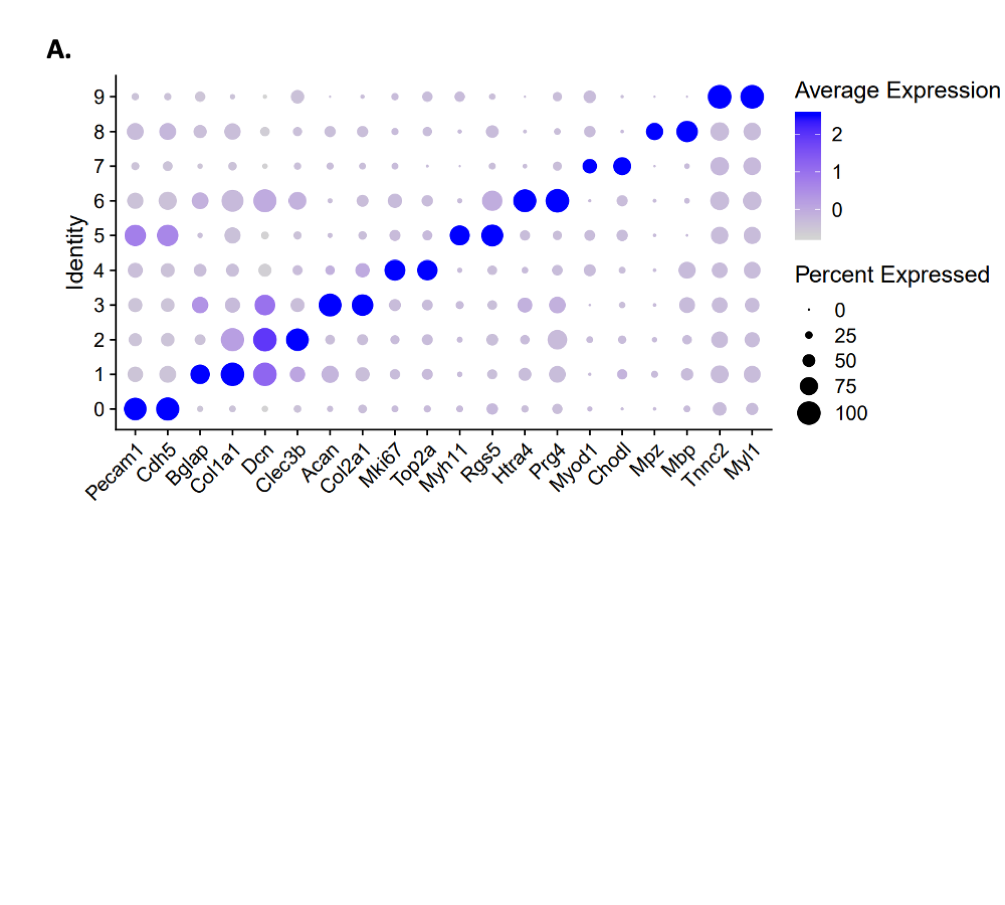
**

**Supp. Figure 3.** **Single cell RNA-seq analysis of the knee joint** showing the expression of selected markers of various cell types**.** (A) Dot size represents the % of cells expressing a specific marker, while the intensity of color indicates the average expression level for that gene, in that cluster.

**Supplementary table 1.** Bulk RNA-seq data from control joints compared to type 1 diabetes mellitus joints. Fold upregulation (log2 scale).

| **Gene** | **Expression** | **Adjusted p-Value** |
| --- | --- | --- |
| Adamts14 | 0.527209504 | 0.010719 |
| Adam11 | 0.531601705 | 0.00089 |
| Adamts10 | 0.554728087 | 2.60E-07 |
| Mmp23 | 0.554991122 | 0.000514 |
| Adamts4 | 0.57262948 | 0.002292 |
| Mmp17 | 0.61010057 | 0.02295 |
| Mmp11 | 0.742591725 | 5.20E-05 |
| Adam33 | 0.854710405 | 8.84E-08 |
| Mmp28 | 0.94006589 | 7.85E-07 |
| Cdkn1c | 0.500342984 | 0.002674 |
| Eif6 | 0.50040379 | 0.000371 |
| Specc1 | 0.500582823 | 0.00011 |
| Rcc2 | 0.500587892 | 0.000112 |
| Rab38 | 0.500639193 | 0.005246 |
| Rspo3 | 0.500787218 | 0.023073 |
| Slc25a39 | 0.501147598 | 4.66E-06 |
| Msl3l2 | 0.50120368 | 0.028352 |
| Wnt10b | 0.501430732 | 0.017439 |
| Gadd45b | 0.501759933 | 0.017093 |
| Eif4ebp1 | 0.50192532 | 1.55E-05 |
| Cd19 | 0.502143989 | 0.014311 |
| Slc8b1 | 0.502747829 | 0.004689 |
| Nfe2 | 0.503031339 | 0.000143 |
| D8Ertd82e | 0.503052209 | 0.008401 |
| Mcm10 | 0.503631153 | 7.74E-05 |
| Agrn | 0.50365489 | 9.78E-06 |
| Ampd2 | 0.50368607 | 3.87E-05 |
| Tle2 | 0.503882711 | 9.47E-05 |
| Timeless | 0.504485042 | 1.30E-05 |
| Aprt | 0.504499964 | 0.003353 |
| Incenp | 0.504549838 | 0.000216 |
| Enkd1 | 0.504751936 | 0.002391 |
| Nfkbie | 0.504797786 | 0.000326 |
| Ormdl2 | 0.505042626 | 0.002283 |
| Myl6b | 0.505141472 | 0.00246 |
| Ddah2 | 0.505325295 | 0.000273 |
| B4galnt3 | 0.505678399 | 0.013417 |
| Ccs | 0.505951891 | 0.007779 |
| P3h4 | 0.506380083 | 9.97E-05 |
| Pik3ap1 | 0.507000023 | 0.000691 |
| Sdk2 | 0.507065144 | 0.017306 |
| Lst1 | 0.507336072 | 0.000226 |
| Fermt3 | 0.507406628 | 1.19E-06 |
| Cdca5 | 0.507534656 | 0.000312 |
| Lax1 | 0.507652697 | 0.019109 |
| Adck5 | 0.507745405 | 0.000847 |
| Slc4a2 | 0.508366401 | 0.000134 |
| Toe1 | 0.508759742 | 0.000175 |
| Ccdc88b | 0.509493301 | 0.000608 |
| Myo1g | 0.509989444 | 0.000136 |
| Galnt10 | 0.510008582 | 0.001032 |
| Pcnx3 | 0.510396783 | 1.01E-06 |
| Dscc1 | 0.510428167 | 0.008902 |
| Bpgm | 0.510454228 | 0.00016 |
| Gpr18 | 0.510584011 | 0.009298 |
| Ifrd2 | 0.510655424 | 0.000149 |
| Ifitm3 | 0.510667164 | 0.000267 |
| Igsf9 | 0.510694415 | 0.036591 |
| H2-M3 | 0.510842273 | 0.000725 |
| Dclk2 | 0.510922807 | 0.018006 |
| Pear1 | 0.510975212 | 4.10E-06 |
| Slc6a13 | 0.511091852 | 0.01348 |
| Adra2a | 0.51142935 | 0.022736 |
| 2700081O15Rik | 0.511484087 | 3.15E-05 |
| Phlpp2 | 0.511912531 | 0.000143 |
| Frs3 | 0.512155972 | 0.004544 |
| BC017158 | 0.51278438 | 0.000132 |
| Csk | 0.512826204 | 1.22E-05 |
| Rgs14 | 0.51301508 | 9.08E-05 |
| Cdca3 | 0.513180246 | 0.000174 |
| Runx3 | 0.513237016 | 0.00056 |
| Zdhhc14 | 0.513352755 | 4.65E-05 |
| C1qtnf6 | 0.513415996 | 0.001503 |
| Mfng | 0.513557227 | 0.000542 |
| Sbk2 | 0.513575012 | 0.01752 |
| Amz1 | 0.513692437 | 0.003604 |
| Col28a1 | 0.513721785 | 0.047116 |
| Trp53i13 | 0.513848153 | 0.000956 |
| Fndc4 | 0.514387141 | 0.006137 |
| Nek2 | 0.514415391 | 0.002391 |
| Ogfr | 0.514417619 | 0.00048 |
| Rrm2 | 0.514701913 | 0.000251 |
| Sh2d2a | 0.515063899 | 0.021123 |
| Snai1 | 0.515158074 | 0.00183 |
| Mfsd10 | 0.515233911 | 4.16E-05 |
| Fscn1 | 0.515422955 | 0.000253 |
| Dusp10 | 0.515449276 | 0.003102 |
| Plpp2 | 0.515576853 | 0.010036 |
| Polg2 | 0.515851299 | 0.010187 |
| Srm | 0.515877596 | 7.22E-05 |
| Tuba1b | 0.516430579 | 5.10E-05 |
| Sema4b | 0.516479788 | 2.96E-05 |
| Mcm7 | 0.516480904 | 1.40E-05 |
| Hes6 | 0.516615065 | 5.24E-05 |
| Tmem9 | 0.516718293 | 4.42E-05 |
| Rabep2 | 0.516768252 | 8.16E-05 |
| Epb42 | 0.516770779 | 0.000564 |
| Arhgap9 | 0.516803049 | 0.000374 |
| Smim4 | 0.51721869 | 0.040641 |
| B4galnt1 | 0.51755449 | 3.25E-05 |
| Tpgs1 | 0.517761085 | 0.000768 |
| Mogs | 0.517914253 | 0.000766 |
| Tnfsf14 | 0.518033301 | 0.000274 |
| Tspyl3 | 0.518520528 | 0.001842 |
| Loxl4 | 0.519077183 | 0.003624 |
| Ranbp10 | 0.519165749 | 1.44E-06 |
| Kif22 | 0.519196958 | 1.96E-05 |
| Atp13a2 | 0.519628814 | 9.96E-06 |
| Camsap3 | 0.519883486 | 0.036953 |
| Cmpk2 | 0.520170107 | 0.000521 |
| Cldn5 | 0.520251061 | 0.035647 |
| Psmb8 | 0.520521782 | 0.000116 |
| Cdk1 | 0.520935358 | 0.00061 |
| St6galnac4 | 0.521000085 | 6.05E-05 |
| Sirt7 | 0.521071312 | 4.79E-05 |
| Phldb3 | 0.52120707 | 0.011076 |
| Nrros | 0.521840021 | 5.10E-05 |
| Ubash3b | 0.522273188 | 2.02E-05 |
| Lpcat1 | 0.522340449 | 0.000752 |
| Zfp296 | 0.522371925 | 0.003144 |
| Cd79b | 0.52311163 | 0.003837 |
| Cad | 0.523193554 | 2.91E-05 |
| Il21r | 0.52347227 | 0.000434 |
| Ap5b1 | 0.523545773 | 0.007649 |
| Slc1a5 | 0.523609511 | 0.00057 |
| Tmem40 | 0.523930247 | 0.00043 |
| Ripk3 | 0.524135044 | 0.00027 |
| Cela1 | 0.52437937 | 0.000346 |
| Pim2 | 0.525056916 | 0.000466 |
| Spef1 | 0.525104458 | 0.002106 |
| Bmp4 | 0.525824922 | 0.00438 |
| H2-Ab1 | 0.525949445 | 2.32E-05 |
| Itgb7 | 0.526152925 | 1.30E-05 |
| Pkhd1l1 | 0.526532659 | 0.011088 |
| Sox10 | 0.526548646 | 0.011562 |
| Unc119 | 0.526657589 | 1.32E-05 |
| Gm7694 | 0.526858195 | 0.000161 |
| Rab43 | 0.527030545 | 0.000368 |
| Gm5617 | 0.52714936 | 0.005753 |
| Wisp2 | 0.527252061 | 0.002569 |
| Ldlrap1 | 0.527611947 | 2.57E-05 |
| Ccdc85b | 0.527624694 | 0.001252 |
| Sertad3 | 0.527942769 | 2.44E-05 |
| Src | 0.527960493 | 7.86E-05 |
| Fam109b | 0.528144958 | 0.001999 |
| Cfp | 0.528377135 | 0.000415 |
| Dnase2a | 0.528436837 | 1.36E-05 |
| Cdc6 | 0.528632636 | 0.000289 |
| Nrgn | 0.528659163 | 0.000443 |
| Card10 | 0.528717321 | 2.16E-05 |
| Tmem163 | 0.528883573 | 0.029506 |
| Impa2 | 0.529829925 | 0.000548 |
| Icosl | 0.530329385 | 1.10E-05 |
| Bcl3 | 0.530438999 | 0.000226 |
| Lgr6 | 0.53066821 | 0.00278 |
| Dnd1 | 0.530867203 | 0.028804 |
| Tmco6 | 0.53114763 | 0.000126 |
| Ackr1 | 0.53122825 | 0.000317 |
| Upb1 | 0.531435757 | 0.034746 |
| Efnb3 | 0.531534419 | 0.010349 |
| Pdzd4 | 0.532126954 | 0.000427 |
| Zbtb49 | 0.532426338 | 0.001475 |
| Tmem8 | 0.53299443 | 0.000197 |
| Xrcc3 | 0.533033738 | 0.000303 |
| Adgrg5 | 0.533049939 | 0.01951 |
| Bax | 0.533050713 | 5.31E-06 |
| Map3k11 | 0.533421141 | 1.86E-05 |
| Fam109a | 0.533432332 | 4.25E-05 |
| Wdr83os | 0.534573795 | 0.011 |
| Cdca4 | 0.535024197 | 0.000195 |
| Rps18 | 0.535192574 | 0.001878 |
| Ctu2 | 0.53539053 | 0.000257 |
| Usp20 | 0.536104868 | 8.58E-05 |
| Pitpnm1 | 0.536220904 | 8.52E-06 |
| Sapcd2 | 0.536274526 | 0.005013 |
| Reep2 | 0.536416068 | 0.007619 |
| Tal1 | 0.536472653 | 0.000397 |
| Asprv1 | 0.536707878 | 0.045504 |
| Tmem79 | 0.537704834 | 0.041526 |
| Snn | 0.537726385 | 0.001038 |
| Tmem86a | 0.53816476 | 2.06E-05 |
| S1pr4 | 0.538663955 | 5.07E-05 |
| Ier5l | 0.538906224 | 0.016343 |
| Ptk2b | 0.539481095 | 8.20E-07 |
| Wdr91 | 0.53969292 | 8.16E-05 |
| Fkbp11 | 0.540593791 | 0.003844 |
| Rcc1 | 0.540868926 | 0.000186 |
| Tspan4 | 0.541135764 | 0.000176 |
| Atpif1 | 0.541473103 | 0.000105 |
| Rhag | 0.541588333 | 0.002296 |
| Traf1 | 0.541719546 | 0.000773 |
| H1fx | 0.542440104 | 0.022928 |
| Cercam | 0.54339202 | 2.72E-05 |
| Wbscr27 | 0.543421361 | 0.000329 |
| Socs3 | 0.54344713 | 0.001678 |
| Stx2 | 0.543766713 | 3.66E-06 |
| Ptch2 | 0.543978883 | 0.017712 |
| Clec14a | 0.544454008 | 0.000212 |
| Lrrc56 | 0.544516828 | 0.001266 |
| Emilin3 | 0.544546419 | 0.034229 |
| Ier3 | 0.544697943 | 0.000231 |
| Mcm3 | 0.545150874 | 0.000123 |
| Sdc3 | 0.545276403 | 4.59E-07 |
| Fam198a | 0.545410477 | 0.012696 |
| Tcirg1 | 0.547475285 | 2.37E-05 |
| Tmem191c | 0.547495098 | 0.004699 |
| Lrrc29 | 0.547536675 | 0.001832 |
| Syne3 | 0.547621752 | 2.21E-05 |
| Plxnb2 | 0.547737477 | 4.33E-07 |
| Dhx34 | 0.548727237 | 1.30E-05 |
| Slc25a38 | 0.548871894 | 0.000122 |
| Tnfaip8l2 | 0.549105661 | 4.54E-06 |
| Ppfia4 | 0.549513787 | 9.80E-06 |
| Sbno2 | 0.549571543 | 4.34E-06 |
| Eme1 | 0.549755222 | 0.000465 |
| Slc6a20b | 0.549775865 | 0.019054 |
| Gimap3 | 0.550270472 | 0.026481 |
| H2afx | 0.550410144 | 0.002668 |
| Cdc25c | 0.551286128 | 0.005261 |
| Hilpda | 0.551366023 | 0.001244 |
| Tubb1 | 0.551512702 | 3.46E-07 |
| Zfp618 | 0.552323936 | 0.042301 |
| Rpl18 | 0.552432994 | 0.000276 |
| Cpt1c | 0.552582859 | 0.000427 |
| Abcb10 | 0.552691497 | 0.000343 |
| Pf4 | 0.553150698 | 0.002977 |
| Unc5b | 0.553300096 | 2.18E-05 |
| Sgsm1 | 0.553563211 | 0.046495 |
| Aatk | 0.553650159 | 0.000193 |
| Actr5 | 0.55383294 | 0.000111 |
| Tmem98 | 0.554426949 | 0.002674 |
| Pyroxd2 | 0.554632392 | 0.000741 |
| Chpf2 | 0.554746578 | 1.86E-05 |
| Tmem160 | 0.555175451 | 0.027432 |
| Abca7 | 0.555444903 | 1.87E-06 |
| Fanca | 0.555582683 | 2.16E-05 |
| Rpl13 | 0.555964939 | 0.001814 |
| Egfl8 | 0.556121212 | 0.002022 |
| Galnt15 | 0.556727333 | 6.61E-05 |
| Kcnq4 | 0.557289038 | 0.006446 |
| Tesc | 0.557552107 | 0.000725 |
| Zbtb42 | 0.558276648 | 0.000137 |
| Atox1 | 0.558407273 | 0.000155 |
| Cstb | 0.560205692 | 3.28E-05 |
| Slpi | 0.560355602 | 0.0079 |
| Cdr2 | 0.560633358 | 0.000388 |
| Inf2 | 0.560647337 | 1.64E-08 |
| Fen1 | 0.562331361 | 9.30E-05 |
| Cenpw | 0.562789417 | 0.004663 |
| Fbp1 | 0.563045522 | 0.046321 |
| Ift140 | 0.563143309 | 8.46E-05 |
| Panx3 | 0.563407835 | 0.04948 |
| Cdc25b | 0.563947572 | 2.41E-05 |
| Efna1 | 0.56433665 | 0.000961 |
| Trim56 | 0.564760727 | 5.75E-06 |
| Col13a1 | 0.565266345 | 0.00011 |
| Ucp2 | 0.565331129 | 3.13E-08 |
| Wdr81 | 0.565742648 | 7.58E-06 |
| Gli1 | 0.565956075 | 0.000156 |
| Mfsd13a | 0.566005457 | 0.001104 |
| Cfd | 0.566121569 | 0.019042 |
| BC021614 | 0.566190962 | 0.025174 |
| Ube2s | 0.566478235 | 0.004044 |
| Slc25a34 | 0.566508593 | 0.000806 |
| Zfp707 | 0.566773737 | 0.001233 |
| Vars | 0.568023025 | 3.21E-05 |
| Unc13d | 0.56862263 | 7.35E-06 |
| Tspo | 0.568624117 | 0.005295 |
| Cass4 | 0.568698424 | 0.001968 |
| Adgra2 | 0.5687425 | 1.00E-05 |
| Rgs12 | 0.56904817 | 8.72E-06 |
| Disc1 | 0.569389158 | 0.033973 |
| Rpl13a | 0.570561095 | 0.000125 |
| Nfatc4 | 0.570666681 | 5.06E-05 |
| Rpgrip1 | 0.571002217 | 0.009947 |
| Frmd8 | 0.571068698 | 0.000312 |
| Nmral1 | 0.571135322 | 0.000349 |
| Snx20 | 0.571305349 | 7.56E-05 |
| Gp5 | 0.571826684 | 1.59E-05 |
| Sema4f | 0.572113431 | 0.002931 |
| Caskin1 | 0.572133782 | 0.037055 |
| Sh2d5 | 0.572456559 | 0.003353 |
| Chaf1b | 0.572833386 | 4.12E-05 |
| Selm | 0.57288562 | 6.59E-06 |
| Hist1h3d | 0.572940191 | 0.008535 |
| Mgat4b | 0.573251494 | 8.30E-07 |
| Art4 | 0.573430913 | 0.000127 |
| Csf1r | 0.573665265 | 6.05E-06 |
| Agfg2 | 0.573685048 | 2.84E-06 |
| Mpp2 | 0.573699224 | 0.000107 |
| Gpr35 | 0.573857386 | 0.001329 |
| Rps14 | 0.574069032 | 0.012038 |
| Haus5 | 0.574194103 | 4.94E-06 |
| Pold1 | 0.574403963 | 3.08E-06 |
| Tmc8 | 0.575548461 | 1.15E-05 |
| Pgpep1l | 0.575769298 | 0.004594 |
| Spns2 | 0.576712611 | 0.000797 |
| Txnrd2 | 0.577131101 | 0.000182 |
| Hdac7 | 0.577181931 | 2.04E-07 |
| Efs | 0.57759236 | 0.001134 |
| Adora2a | 0.578132717 | 0.000493 |
| Nme3 | 0.579118004 | 0.000143 |
| Nrip3 | 0.579215061 | 0.013345 |
| Nphp4 | 0.579279543 | 0.016108 |
| Fbp2 | 0.579586015 | 0.005909 |
| Hgh1 | 0.579681074 | 0.000152 |
| Ly6g6f | 0.579838182 | 0.000318 |
| Card11 | 0.580438306 | 3.58E-05 |
| Svbp | 0.580527075 | 6.28E-05 |
| Zfp105 | 0.580547017 | 0.018925 |
| Slc2a9 | 0.580784949 | 0.006734 |
| Ankrd54 | 0.580882077 | 3.97E-06 |
| Slc22a17 | 0.582784298 | 0.000409 |
| Ldb1 | 0.582904573 | 2.02E-08 |
| H2-T24 | 0.583181554 | 7.77E-07 |
| Sytl1 | 0.583417495 | 0.0029 |
| Pld4 | 0.583478173 | 5.11E-07 |
| Espl1 | 0.583987952 | 0.000269 |
| Wtip | 0.583994234 | 0.000133 |
| Ttyh3 | 0.584140587 | 1.30E-05 |
| Prrx2 | 0.584315221 | 0.019211 |
| Metrnl | 0.584408466 | 6.43E-07 |
| Zfp503 | 0.584493486 | 0.001992 |
| Arhgap23 | 0.584505267 | 1.14E-06 |
| Nt5c | 0.584658988 | 1.30E-05 |
| Iffo1 | 0.58514362 | 1.70E-06 |
| Slc37a2 | 0.586128518 | 7.88E-05 |
| Angptl4 | 0.586358004 | 0.00171 |
| Tmprss3 | 0.586450989 | 0.003808 |
| Plk1 | 0.586505407 | 2.66E-05 |
| Gm8909 | 0.586595887 | 0.001982 |
| Rasal3 | 0.586657488 | 5.57E-06 |
| Plppr3 | 0.586765487 | 5.72E-05 |
| Adora3 | 0.586961421 | 0.005316 |
| Tmem176a | 0.587846284 | 1.50E-05 |
| 4930427A07Rik | 0.5890259 | 0.002213 |
| Ces2g | 0.589191882 | 1.97E-05 |
| Zfp385a | 0.589225055 | 1.71E-06 |
| Sox18 | 0.589397126 | 1.45E-05 |
| Arsi | 0.589686344 | 0.009554 |
| Gm15800 | 0.589981107 | 2.49E-07 |
| Fam69b | 0.59004891 | 0.003427 |
| Aplnr | 0.590277465 | 0.00042 |
| Kifc5b | 0.59031363 | 5.45E-06 |
| Zfyve28 | 0.59040662 | 0.0026 |
| Rem1 | 0.591396337 | 0.003459 |
| Asb6 | 0.591574987 | 1.07E-05 |
| Mcm5 | 0.591622056 | 4.46E-06 |
| Kif26a | 0.592473508 | 9.55E-05 |
| Eps8l1 | 0.592730032 | 0.033834 |
| Gp1bb | 0.592788313 | 4.48E-07 |
| Slamf9 | 0.592897808 | 0.002148 |
| Limk1 | 0.59292203 | 1.40E-05 |
| Slc25a10 | 0.593121859 | 0.000201 |
| Meis3 | 0.593351578 | 0.000346 |
| Lrrc45 | 0.593513534 | 2.55E-06 |
| Tlcd2 | 0.593539353 | 0.004418 |
| Ube2o | 0.593553193 | 1.66E-06 |
| Ccl8 | 0.593711418 | 0.035686 |
| Ermap | 0.594383618 | 3.19E-05 |
| Bambi | 0.594711806 | 5.21E-05 |
| Prdx2 | 0.595140639 | 2.16E-05 |
| Gm14137 | 0.596070418 | 0.012243 |
| Cdc42bpg | 0.596380313 | 3.52E-05 |
| Rnd2 | 0.596772369 | 0.003141 |
| Shank1 | 0.597152262 | 0.022816 |
| Agpat4 | 0.597204041 | 1.34E-06 |
| Tnfrsf11a | 0.597336093 | 0.001767 |
| Iqcd | 0.598864512 | 0.034501 |
| Sppl2b | 0.59921354 | 4.48E-07 |
| Ssbp4 | 0.599379505 | 3.42E-06 |
| Cbx2 | 0.599773096 | 0.000781 |
| Cnnm1 | 0.600204408 | 0.016663 |
| Tmem132e | 0.600718961 | 0.025213 |
| Cacna1g | 0.600946442 | 0.000148 |
| Prss34 | 0.601146192 | 1.09E-05 |
| Klhl36 | 0.601284174 | 3.28E-05 |
| Ttc39a | 0.601490072 | 0.031738 |
| Cd7 | 0.602048695 | 0.000326 |
| Engase | 0.602706406 | 6.12E-05 |
| Klf16 | 0.60300652 | 4.77E-05 |
| Galnt9 | 0.603551837 | 0.003763 |
| Nckap5l | 0.604784619 | 0.000107 |
| S1pr2 | 0.605003713 | 2.95E-05 |
| Dbndd1 | 0.605388237 | 0.003979 |
| 2310011J03Rik | 0.605967481 | 0.000579 |
| Taf1c | 0.608874718 | 5.15E-06 |
| Atp7b | 0.608889214 | 0.005164 |
| Gimap1 | 0.610301251 | 1.64E-05 |
| Id3 | 0.610596825 | 4.88E-07 |
| Rnf213 | 0.610945645 | 6.03E-09 |
| Slc43a1 | 0.611069576 | 6.81E-09 |
| Coro1a | 0.611072988 | 0.00063 |
| Syce2 | 0.611168091 | 0.000248 |
| Necab3 | 0.611179967 | 0.020502 |
| Pth1r | 0.611418579 | 9.86E-06 |
| Pqlc1 | 0.611634164 | 0.000904 |
| Adar | 0.611883321 | 2.06E-08 |
| Slc25a22 | 0.612890192 | 3.16E-05 |
| Mid1 | 0.613021827 | 0.003454 |
| Cdc20 | 0.613892542 | 1.53E-05 |
| Bcl7c | 0.614623124 | 0.000248 |
| Ptpn18 | 0.614921175 | 6.99E-05 |
| Pafah1b3 | 0.615003711 | 0.000491 |
| Oaz1 | 0.615960668 | 0.001326 |
| Chst2 | 0.616279889 | 0.000121 |
| Tnfrsf14 | 0.616837093 | 2.94E-05 |
| Dok3 | 0.617300838 | 3.57E-06 |
| BC030867 | 0.617836291 | 0.000188 |
| Klhl25 | 0.617970708 | 1.61E-05 |
| Tmem158 | 0.618205747 | 0.000592 |
| Colec11 | 0.618751643 | 0.046657 |
| Scand1 | 0.619777333 | 0.009578 |
| Col27a1 | 0.619970546 | 8.23E-05 |
| Irf8 | 0.619990727 | 2.72E-06 |
| Hivep3 | 0.620070536 | 0.000408 |
| Slfn14 | 0.620093659 | 1.10E-05 |
| Lrwd1 | 0.620257901 | 2.05E-08 |
| Aldh16a1 | 0.620529193 | 3.08E-05 |
| Phf11b | 0.620656532 | 0.001564 |
| Azin2 | 0.620757604 | 0.004468 |
| Itga2b | 0.621570583 | 3.01E-06 |
| Tsku | 0.62308257 | 1.52E-05 |
| Lig1 | 0.623167971 | 4.54E-08 |
| Btnl2 | 0.623248895 | 0.009314 |
| Gata1 | 0.624130008 | 0.000113 |
| Clec10a | 0.624170254 | 9.86E-06 |
| Ccdc180 | 0.626074957 | 0.01697 |
| Plekhg4 | 0.62618905 | 0.000667 |
| Doc2g | 0.626703658 | 0.000679 |
| Fam26f | 0.626756176 | 0.010106 |
| Avpr2 | 0.626925789 | 0.026257 |
| H2afz | 0.62843538 | 0.000219 |
| Eml3 | 0.628972185 | 1.07E-07 |
| Slc6a20a | 0.629037293 | 0.000329 |
| Cchcr1 | 0.629402982 | 3.55E-05 |
| Mkrn1 | 0.629431839 | 7.40E-06 |
| Unc5cl | 0.62946774 | 0.004196 |
| Cpne7 | 0.629867304 | 0.035078 |
| Sh3tc1 | 0.62987192 | 1.02E-05 |
| Tlcd1 | 0.630070374 | 2.17E-05 |
| Cd40 | 0.630599995 | 0.000176 |
| Smim6 | 0.631597278 | 0.017694 |
| Slc13a5 | 0.632078427 | 0.004482 |
| Cacnb3 | 0.632272762 | 0.000425 |
| Spon2 | 0.632534193 | 0.005662 |
| Rps19 | 0.632797868 | 0.000235 |
| Phyhip | 0.632833227 | 0.000171 |
| Map4k1 | 0.633479249 | 1.35E-07 |
| Adrb3 | 0.633637533 | 0.031265 |
| Snx15 | 0.634017458 | 1.39E-05 |
| Tbxa2r | 0.634310554 | 5.72E-05 |
| Myl4 | 0.634709661 | 0.000168 |
| Psrc1 | 0.635103707 | 8.64E-05 |
| Nrg2 | 0.635129652 | 0.009943 |
| Zbp1 | 0.635362367 | 9.22E-05 |
| Fbxl6 | 0.636243433 | 8.27E-05 |
| C1qc | 0.637004737 | 2.61E-06 |
| Fzr1 | 0.637041795 | 5.47E-10 |
| Cabin1 | 0.637530265 | 1.46E-10 |
| Pax5 | 0.637629833 | 0.000346 |
| Col1a2 | 0.63792591 | 3.93E-07 |
| Cilp2 | 0.638254748 | 0.000326 |
| Fbxl15 | 0.638753463 | 0.005244 |
| Bdh2 | 0.638785184 | 0.010265 |
| Chaf1a | 0.638863681 | 7.08E-06 |
| Gja4 | 0.639770094 | 0.00024 |
| Slc27a1 | 0.639868755 | 1.07E-06 |
| Npas4 | 0.640794786 | 0.00066 |
| Gm996 | 0.641192889 | 0.004987 |
| Msi1 | 0.641205089 | 0.015118 |
| Alad | 0.641530888 | 1.05E-05 |
| H2-Eb1 | 0.64163542 | 2.67E-05 |
| Ly6g5b | 0.642042275 | 0.004496 |
| Recql4 | 0.642344938 | 5.99E-07 |
| Grap2 | 0.642454849 | 3.36E-05 |
| Hic1 | 0.642463867 | 0.000158 |
| Ifitm5 | 0.642518473 | 0.000221 |
| Dact3 | 0.642902457 | 0.000139 |
| Pigq | 0.644096212 | 5.23E-06 |
| Peg3 | 0.645081922 | 0.000216 |
| Homer3 | 0.645347954 | 1.80E-06 |
| Mdk | 0.645649332 | 4.44E-05 |
| Pask | 0.645797523 | 1.21E-05 |
| Gale | 0.646005345 | 9.91E-06 |
| Myo1a | 0.646646702 | 0.040915 |
| Prr22 | 0.646771224 | 0.037174 |
| Sigirr | 0.646990107 | 1.79E-05 |
| Cd3d | 0.647824565 | 0.005448 |
| Sox12 | 0.647830812 | 2.00E-05 |
| Adap1 | 0.647900037 | 1.50E-05 |
| Cenpm | 0.648619189 | 0.000189 |
| Cd22 | 0.648870422 | 0.00204 |
| Ccdc92 | 0.649397265 | 0.000904 |
| Angpt4 | 0.649454368 | 0.002017 |
| 2610524H06Rik | 0.649479279 | 0.001622 |
| Sox4 | 0.649873401 | 4.78E-06 |
| Kcnk5 | 0.649883743 | 0.000235 |
| Sowaha | 0.649892355 | 1.59E-05 |
| Siglecg | 0.649989411 | 8.09E-06 |
| Nudt11 | 0.650411579 | 0.022815 |
| Sp7 | 0.65048009 | 0.000488 |
| Ces2e | 0.650631187 | 0.048543 |
| Mrm1 | 0.650733607 | 7.66E-05 |
| Gm9733 | 0.650817472 | 0.001778 |
| Sele | 0.651218068 | 0.001068 |
| Asns | 0.651849576 | 8.11E-06 |
| Fam117a | 0.652504022 | 1.10E-07 |
| AA986860 | 0.652838326 | 0.004207 |
| Mesdc1 | 0.653191404 | 9.68E-05 |
| Lyl1 | 0.653286476 | 8.52E-07 |
| Pml | 0.653510101 | 2.19E-10 |
| Dedd2 | 0.653761596 | 3.45E-06 |
| Haao | 0.653840647 | 4.29E-06 |
| Pbx4 | 0.655147437 | 0.011025 |
| Rps29 | 0.655220637 | 0.019089 |
| Adcy3 | 0.655230815 | 6.59E-06 |
| Tcf3 | 0.655767582 | 2.72E-08 |
| Tnfrsf13b | 0.656549723 | 0.001467 |
| Myo1h | 0.656615089 | 0.015372 |
| Cox6b2 | 0.657846092 | 3.59E-05 |
| Snrpg | 0.657939653 | 0.000566 |
| Ppp1r15a | 0.658017186 | 6.17E-10 |
| Ccr7 | 0.658096225 | 0.000583 |
| Lfng | 0.658176826 | 3.19E-06 |
| H2afj | 0.658603751 | 5.68E-05 |
| Trappc6a | 0.659340877 | 9.16E-05 |
| Tns4 | 0.660410002 | 0.008764 |
| Gm590 | 0.66205086 | 0.049903 |
| Ankle1 | 0.662298605 | 0.000114 |
| Lamc3 | 0.663329179 | 0.000306 |
| Sh2d1b1 | 0.663489318 | 0.001599 |
| B3gnt8 | 0.664567701 | 1.27E-05 |
| Nrtn | 0.665013425 | 0.013545 |
| Clic3 | 0.665132235 | 0.006763 |
| 1700027J19Rik | 0.665903413 | 0.000865 |
| Xaf1 | 0.666208397 | 3.86E-06 |
| Ccdc166 | 0.666712541 | 0.002861 |
| Vill | 0.667429867 | 0.000282 |
| Basp1 | 0.667536348 | 0.001382 |
| Col11a2 | 0.668271611 | 2.92E-06 |
| Fzd10 | 0.668371094 | 0.024203 |
| Sh3tc2 | 0.668656229 | 0.000121 |
| Penk | 0.668904994 | 1.98E-05 |
| Bglap2 | 0.669015742 | 1.93E-05 |
| Lck | 0.669119856 | 0.000107 |
| Apc2 | 0.669289698 | 0.022872 |
| Capn5 | 0.669629022 | 5.82E-08 |
| Pabpc1l | 0.669889307 | 0.003352 |
| Col2a1 | 0.669899248 | 0.003075 |
| Podnl1 | 0.670536163 | 1.00E-05 |
| Dnajb3 | 0.671159576 | 0.0034 |
| Rnaseh2c | 0.671386943 | 0.007883 |
| Fam212a | 0.671393522 | 3.55E-05 |
| Ltb4r2 | 0.671697648 | 0.036285 |
| Crtac1 | 0.671899044 | 0.004763 |
| Ciita | 0.672003154 | 0.002317 |
| Gnaz | 0.672173689 | 1.94E-05 |
| Rltpr | 0.672579954 | 5.17E-06 |
| Bst2 | 0.672623077 | 4.56E-08 |
| Ffar4 | 0.673115849 | 0.024325 |
| Foxs1 | 0.673701411 | 0.001888 |
| Cdhr4 | 0.674522348 | 0.001317 |
| Wscd2 | 0.67534657 | 0.001567 |
| Nectin4 | 0.675386242 | 0.0047 |
| Pycr2 | 0.675897167 | 1.62E-06 |
| Gpc2 | 0.676416916 | 5.61E-05 |
| Glis2 | 0.677590398 | 1.51E-05 |
| A230050P20Rik | 0.67829494 | 8.21E-06 |
| E430018J23Rik | 0.678944891 | 0.000253 |
| Apoe | 0.67950906 | 1.17E-07 |
| Ptprcap | 0.679683229 | 6.25E-06 |
| Emilin1 | 0.680069163 | 7.61E-08 |
| Six2 | 0.680321011 | 0.001394 |
| Fam65c | 0.680616885 | 2.02E-07 |
| Galk1 | 0.68081685 | 4.02E-05 |
| Ier5 | 0.681488026 | 5.70E-07 |
| Pigz | 0.681838398 | 0.014538 |
| Ctsk | 0.683104059 | 3.42E-05 |
| Gm266 | 0.68417286 | 0.044135 |
| Col10a1 | 0.684490413 | 0.01182 |
| Dok1 | 0.68472557 | 1.91E-05 |
| Lif | 0.686171593 | 0.001315 |
| Tmcc2 | 0.686625594 | 7.00E-06 |
| Kel | 0.686772151 | 7.13E-06 |
| Pnpo | 0.686797319 | 6.55E-08 |
| Zfp688 | 0.68708511 | 0.000158 |
| Pcsk4 | 0.687133507 | 0.002317 |
| Slc38a5 | 0.687505746 | 1.72E-05 |
| Tbx6 | 0.688500345 | 2.21E-05 |
| Anxa9 | 0.688507118 | 0.000205 |
| Grik5 | 0.688683092 | 7.51E-08 |
| BC022687 | 0.689579815 | 0.014309 |
| Tlr12 | 0.689869128 | 0.000383 |
| Gls2 | 0.690163346 | 0.017617 |
| Oaf | 0.690915693 | 1.18E-05 |
| Psd2 | 0.691085386 | 0.027133 |
| Fcna | 0.691145597 | 2.63E-07 |
| Chst7 | 0.692177112 | 0.000802 |
| Chtf18 | 0.692813446 | 0.000128 |
| 9-Mar | 0.694066616 | 0.007545 |
| Evpl | 0.69420529 | 0.005626 |
| Hsh2d | 0.694909455 | 0.000175 |
| Baiap3 | 0.695110603 | 0.000206 |
| 1300017J02Rik | 0.695625574 | 2.08E-05 |
| Pck2 | 0.696089245 | 3.70E-06 |
| 4930404N11Rik | 0.696883761 | 1.64E-05 |
| Clec4g | 0.697822053 | 0.017671 |
| Sphk1 | 0.698215474 | 1.35E-05 |
| Guca1a | 0.698299572 | 0.041295 |
| Baiap2l2 | 0.698372624 | 0.005508 |
| Isyna1 | 0.698778033 | 5.39E-08 |
| Dpep2 | 0.698919163 | 0.000346 |
| Clcn2 | 0.698981141 | 1.36E-06 |
| Oas1b | 0.699566594 | 1.42E-05 |
| Smim5 | 0.701346643 | 1.80E-06 |
| Lat | 0.701699539 | 1.91E-05 |
| Ppp1r35 | 0.702921018 | 0.004329 |
| Pidd1 | 0.702961785 | 2.49E-07 |
| Fam167b | 0.703198035 | 0.000384 |
| Orc1 | 0.703227323 | 1.02E-05 |
| Phf11a | 0.703248614 | 0.004038 |
| Slc27a3 | 0.7035739 | 3.21E-05 |
| Mt3 | 0.70360606 | 0.000695 |
| Cox6a1 | 0.704409016 | 0.000165 |
| Pkmyt1 | 0.706335549 | 2.94E-06 |
| Matk | 0.707926754 | 7.59E-07 |
| Ccl5 | 0.709151375 | 3.70E-05 |
| Bik | 0.709902299 | 0.007873 |
| Nlgn3 | 0.712949785 | 0.009038 |
| Nek8 | 0.713432257 | 2.25E-07 |
| Kcnk12 | 0.713869623 | 0.004801 |
| Pax7 | 0.713942497 | 0.005836 |
| Fgd2 | 0.713943739 | 1.76E-08 |
| Eno2 | 0.714389312 | 0.033165 |
| Nhlrc4 | 0.714477492 | 0.020713 |
| Tonsl | 0.714939756 | 1.38E-08 |
| Col4a6 | 0.715469541 | 0.045161 |
| Fcer2a | 0.716236566 | 0.011486 |
| Trmo | 0.716899802 | 0.000631 |
| Ccne1 | 0.717170562 | 7.07E-07 |
| Bsn | 0.717519979 | 1.01E-07 |
| Igfbp2 | 0.717558219 | 0.026852 |
| Fam83f | 0.718228677 | 0.032342 |
| Prdm11 | 0.718664934 | 0.037432 |
| Sp6 | 0.719011385 | 0.002325 |
| Insl3 | 0.719028805 | 0.034567 |
| Col1a1 | 0.719848317 | 1.87E-09 |
| Cyp4f39 | 0.721409485 | 0.008896 |
| Lgals3bp | 0.721429494 | 5.33E-07 |
| Hebp1 | 0.722121937 | 2.43E-06 |
| Mdfi | 0.723712724 | 1.48E-05 |
| Rassf7 | 0.723881317 | 0.00017 |
| Rsad2 | 0.724168579 | 1.47E-06 |
| Car1 | 0.72449387 | 4.90E-07 |
| Tk1 | 0.724880123 | 4.58E-07 |
| Oas3 | 0.725056142 | 6.17E-05 |
| Galnt6 | 0.7251361 | 9.34E-07 |
| H2-Oa | 0.725885314 | 0.000329 |
| Dmtn | 0.727439975 | 5.82E-07 |
| Mfsd12 | 0.728204356 | 4.77E-06 |
| Mblac1 | 0.728484968 | 0.002745 |
| Aunip | 0.7286147 | 3.49E-05 |
| Wdr90 | 0.728654991 | 8.68E-06 |
| E2f2 | 0.728890543 | 6.41E-09 |
| E2f1 | 0.730343474 | 2.49E-06 |
| Itgad | 0.73106727 | 2.67E-05 |
| Il3ra | 0.731478252 | 7.97E-08 |
| Col9a1 | 0.732029021 | 0.009075 |
| Snca | 0.734123426 | 2.51E-05 |
| Mast1 | 0.734796006 | 7.72E-06 |
| Msh5 | 0.735055586 | 0.00021 |
| Usp18 | 0.737846752 | 3.73E-06 |
| Plin5 | 0.73807782 | 6.58E-05 |
| Zfpm1 | 0.738162694 | 1.53E-07 |
| BC048679 | 0.738238229 | 0.001959 |
| Frem2 | 0.738457084 | 0.016923 |
| Mx2 | 0.739687712 | 0.000119 |
| Cd209d | 0.740091451 | 0.010004 |
| Tmc6 | 0.740094921 | 6.06E-08 |
| Gfi1b | 0.742872165 | 1.17E-06 |
| Acsbg1 | 0.742882233 | 0.030719 |
| Dhx58 | 0.74312358 | 7.11E-08 |
| Spag4 | 0.743629664 | 0.031056 |
| Snx22 | 0.743747711 | 3.73E-06 |
| Ltb | 0.744355287 | 1.47E-08 |
| Mxd3 | 0.744733137 | 1.11E-06 |
| Dmkn | 0.744931927 | 0.005738 |
| Traf4 | 0.744962641 | 4.77E-09 |
| Rtp4 | 0.746549744 | 1.50E-05 |
| Ppox | 0.747784382 | 1.81E-09 |
| Add2 | 0.747961315 | 1.81E-06 |
| Zfp647 | 0.747961583 | 0.001833 |
| Chadl | 0.748321944 | 0.001048 |
| Lpar5 | 0.748973765 | 0.000321 |
| Ppp1r1a | 0.750759281 | 6.71E-05 |
| Cd6 | 0.75089812 | 0.002555 |
| Dlk1 | 0.752081585 | 0.021502 |
| Slc7a5 | 0.752197713 | 8.52E-07 |
| Vangl2 | 0.753049106 | 0.002748 |
| Ltk | 0.753606696 | 0.013462 |
| Gp9 | 0.754232071 | 2.36E-05 |
| Kcnd1 | 0.75460748 | 0.001994 |
| Cacna1i | 0.755382411 | 0.000584 |
| Pappa2 | 0.756384018 | 0.002166 |
| Gdf3 | 0.756488541 | 0.013368 |
| Slco4a1 | 0.757123774 | 9.51E-06 |
| Mybl2 | 0.75723808 | 1.04E-08 |
| Kcng1 | 0.757858011 | 0.037625 |
| Eva1b | 0.758041985 | 8.51E-07 |
| 3110079O15Rik | 0.758876394 | 5.96E-05 |
| Rassf10 | 0.759566618 | 0.017713 |
| Slc22a20 | 0.759836357 | 0.005392 |
| Arhgap33 | 0.760212465 | 3.29E-09 |
| Cx3cr1 | 0.760490983 | 2.90E-05 |
| Hoxb6 | 0.761029603 | 0.005548 |
| Fcgr1 | 0.762405906 | 2.83E-06 |
| Lrrc16b | 0.762985121 | 0.003463 |
| Fam46c | 0.763056001 | 4.93E-05 |
| C130050O18Rik | 0.763075501 | 7.57E-07 |
| Rsph9 | 0.763329693 | 0.00289 |
| H2-Bl | 0.763547707 | 0.008636 |
| Snph | 0.765271224 | 0.000276 |
| Hoxb7 | 0.765896993 | 0.005018 |
| Fcho1 | 0.766653677 | 1.22E-09 |
| Serpina3b | 0.767908831 | 0.000584 |
| Hcst | 0.768253601 | 9.30E-05 |
| Fam69c | 0.768845802 | 0.025028 |
| Ptk7 | 0.769729643 | 6.83E-06 |
| Dtx1 | 0.769864784 | 4.19E-05 |
| Sidt1 | 0.770235983 | 0.00083 |
| Sdc1 | 0.771613151 | 4.44E-09 |
| Bbc3 | 0.771634632 | 2.52E-05 |
| Smim1 | 0.772110211 | 9.15E-08 |
| Slc13a3 | 0.773857088 | 0.01117 |
| Apol8 | 0.776484374 | 8.36E-06 |
| Hmbs | 0.777325978 | 4.46E-06 |
| Cidea | 0.777603217 | 0.0115 |
| Cd8a | 0.778529345 | 0.000359 |
| Palm2 | 0.779062037 | 0.023571 |
| Mst1r | 0.780206391 | 5.07E-10 |
| Scarf2 | 0.78075029 | 2.52E-08 |
| Cd96 | 0.78077482 | 0.015751 |
| 9930012K11Rik | 0.781397513 | 0.000554 |
| Zcchc3 | 0.786178481 | 1.27E-06 |
| Mfap2 | 0.787485313 | 1.75E-05 |
| Sspo | 0.791594874 | 0.000718 |
| Fjx1 | 0.791876576 | 0.000725 |
| Serpina12 | 0.791966019 | 0.013941 |
| Mroh8 | 0.794679772 | 0.027062 |
| Tspan33 | 0.795642795 | 3.11E-08 |
| Draxin | 0.796588457 | 5.31E-06 |
| Padi3 | 0.800224496 | 0.037546 |
| Mc5r | 0.800470156 | 0.004835 |
| Trib3 | 0.801130995 | 0.00295 |
| E130012A19Rik | 0.80219879 | 0.001656 |
| Sapcd1 | 0.803982929 | 0.019555 |
| Slc9b2 | 0.804740947 | 3.90E-06 |
| Plek2 | 0.805546096 | 0.000958 |
| Tmem119 | 0.805791243 | 5.45E-10 |
| Aoc2 | 0.806651247 | 3.03E-09 |
| Tyro3 | 0.806670463 | 0.000981 |
| Pdia2 | 0.806771567 | 0.000269 |
| G6pd2 | 0.806881915 | 0.002458 |
| Dyrk3 | 0.807518435 | 3.50E-06 |
| Clec4b1 | 0.80796741 | 0.000181 |
| Oas2 | 0.811222293 | 1.18E-07 |
| Slc4a1 | 0.811998742 | 7.85E-07 |
| Cdt1 | 0.812659919 | 1.49E-08 |
| Fam132a | 0.813309734 | 5.85E-07 |
| Gm5134 | 0.814259048 | 0.030123 |
| Adra2c | 0.814699593 | 0.007739 |
| Slc6a12 | 0.815484108 | 7.22E-06 |
| Pdzk1ip1 | 0.815995877 | 0.000107 |
| Krt80 | 0.817106809 | 8.66E-09 |
| Samd14 | 0.817832443 | 4.39E-11 |
| Tjp3 | 0.819525888 | 2.29E-05 |
| Exoc3l4 | 0.820124006 | 2.18E-09 |
| Samd11 | 0.820151891 | 1.37E-08 |
| Slc6a9 | 0.821886772 | 5.47E-10 |
| Ifi44 | 0.824236137 | 0.000919 |
| Notum | 0.827025709 | 0.000705 |
| Msx1 | 0.827560388 | 0.004418 |
| Nyap1 | 0.828188296 | 0.000114 |
| Gng8 | 0.828286138 | 0.045498 |
| Cd247 | 0.829479212 | 7.97E-05 |
| Tmem240 | 0.830437431 | 0.047373 |
| Cd5l | 0.830552121 | 1.51E-08 |
| Mcpt4 | 0.832497271 | 0.000722 |
| Rundc3a | 0.833870438 | 0.000295 |
| Ak8 | 0.83488473 | 0.0131 |
| Tmem51 | 0.835167639 | 3.02E-09 |
| Sdsl | 0.836359282 | 0.000114 |
| Rab3il1 | 0.837100081 | 9.39E-10 |
| Pkp3 | 0.838874455 | 1.31E-05 |
| Gata6 | 0.838875144 | 0.037317 |
| Gpr27 | 0.840536617 | 0.000167 |
| Gbx2 | 0.840634547 | 0.003213 |
| Col9a3 | 0.843148617 | 0.000359 |
| Kcng2 | 0.845522973 | 0.001797 |
| Plxnb1 | 0.845933188 | 1.54E-08 |
| Phf24 | 0.846019001 | 0.011655 |
| Gpx1 | 0.846293959 | 2.98E-08 |
| Hist3h2ba | 0.846561197 | 0.000262 |
| Lrrc25 | 0.846799872 | 2.50E-11 |
| Qrfp | 0.846830189 | 0.043985 |
| Ccdc92b | 0.847956077 | 3.25E-06 |
| Ifit1 | 0.851735003 | 5.17E-06 |
| Trim10 | 0.851941943 | 2.01E-06 |
| Bok | 0.853321805 | 2.98E-07 |
| Fcrl5 | 0.856295882 | 0.049228 |
| Prr7 | 0.858410133 | 2.61E-05 |
| Btn1a1 | 0.858646225 | 0.048797 |
| 6030468B19Rik | 0.859988138 | 3.87E-05 |
| Ebf4 | 0.860038047 | 0.002231 |
| Sit1 | 0.860757287 | 0.007425 |
| Gpr68 | 0.861085415 | 6.51E-06 |
| Ephb3 | 0.86329873 | 3.86E-06 |
| Rhod | 0.866736251 | 5.11E-05 |
| Tnni1 | 0.867970018 | 0.000106 |
| Abcg4 | 0.869036472 | 1.11E-05 |
| Klf1 | 0.870751266 | 2.61E-06 |
| Rcor2 | 0.87087448 | 1.35E-07 |
| Fhdc1 | 0.872362051 | 2.45E-08 |
| Uba7 | 0.872959489 | 2.28E-10 |
| Ninl | 0.873556907 | 1.43E-08 |
| Nrn1l | 0.874273613 | 0.022498 |
| Dusp26 | 0.877137188 | 1.83E-15 |
| Krt18 | 0.877767701 | 0.019225 |
| Gins2 | 0.88130167 | 7.77E-08 |
| Gstt2 | 0.881566116 | 2.23E-07 |
| Wdr86 | 0.883697379 | 2.55E-05 |
| Sec14l2 | 0.884519225 | 1.30E-08 |
| Tspo2 | 0.884882503 | 4.47E-09 |
| Col9a2 | 0.887350298 | 5.23E-05 |
| Paqr6 | 0.887964998 | 0.006593 |
| Hap1 | 0.890373691 | 0.000148 |
| Tigd3 | 0.893104864 | 4.44E-05 |
| Cpne5 | 0.893366504 | 0.02616 |
| Micall2 | 0.896196521 | 1.55E-10 |
| Prss46 | 0.896724457 | 0.017345 |
| Mia | 0.896934713 | 1.08E-05 |
| Gfap | 0.898368407 | 0.000107 |
| Cd8b1 | 0.89840062 | 0.009918 |
| 2610528J11Rik | 0.899859752 | 0.011341 |
| Gjc2 | 0.901764242 | 0.029102 |
| Isg20 | 0.903211074 | 1.12E-11 |
| Jph3 | 0.90327134 | 0.046657 |
| Omp | 0.904103562 | 0.002864 |
| Icam4 | 0.905551388 | 1.93E-07 |
| Pitx1 | 0.907113491 | 1.80E-06 |
| Kcnn4 | 0.907307281 | 1.21E-08 |
| Fbxo2 | 0.911024335 | 0.005611 |
| Slc39a4 | 0.911913157 | 0.000225 |
| Tm4sf19 | 0.914049956 | 2.53E-05 |
| Rhd | 0.915055283 | 9.86E-05 |
| Ngfr | 0.915233579 | 0.001438 |
| Artn | 0.915690163 | 0.000638 |
| Blvrb | 0.918923706 | 5.55E-08 |
| Ckb | 0.920073433 | 3.76E-11 |
| Oscar | 0.920865798 | 1.15E-08 |
| C1qb | 0.924161508 | 0.000434 |
| Tssk6 | 0.924534549 | 0.003421 |
| Tfr2 | 0.927355451 | 2.99E-13 |
| Mfsd6l | 0.927432231 | 0.017354 |
| Alas2 | 0.929165707 | 4.67E-09 |
| Lrrc3 | 0.93146126 | 0.002739 |
| Prss50 | 0.932202968 | 0.000112 |
| Ccr6 | 0.934363441 | 0.001253 |
| Tpsb2 | 0.936091617 | 0.000244 |
| Nmb | 0.938173003 | 1.22E-07 |
| Acp5 | 0.941975536 | 2.17E-09 |
| Oasl2 | 0.948609128 | 1.05E-07 |
| Hyal3 | 0.950726618 | 0.000348 |
| Tac2 | 0.950926547 | 6.36E-05 |
| Tnk1 | 0.951610806 | 0.007236 |
| Adcyap1r1 | 0.955289851 | 1.80E-06 |
| Cd209g | 0.958561126 | 0.000152 |
| Mst1 | 0.95927328 | 1.50E-05 |
| Tmem151b | 0.960703162 | 0.000294 |
| Ypel4 | 0.962535814 | 1.28E-09 |
| Fgf6 | 0.965070662 | 9.18E-05 |
| Susd3 | 0.966048081 | 6.98E-09 |
| Adat3 | 0.966279723 | 0.001956 |
| Hoxb9 | 0.977523511 | 0.004121 |
| Siglec1 | 0.978341511 | 1.36E-09 |
| Rtbdn | 0.980636871 | 0.015349 |
| Trim58 | 0.983223731 | 7.82E-10 |
| Nppc | 0.984694786 | 0.013272 |
| Fgf3 | 0.992051553 | 0.006385 |
| Epor | 0.994800291 | 2.64E-08 |
| Krt7 | 0.995815571 | 0.000136 |
| Cldn13 | 1.001926106 | 6.49E-07 |
| Ptprn2 | 1.002013842 | 0.032425 |
| Qprt | 1.004080113 | 0.001627 |
| 1700029I15Rik | 1.005130274 | 0.007469 |
| Hist1h2ae | 1.00977218 | 0.003102 |
| Dgki | 1.01412017 | 7.94E-05 |
| S1pr5 | 1.019513623 | 2.49E-07 |
| Hba-a1 | 1.025026163 | 7.08E-11 |
| Ocstamp | 1.025838252 | 7.10E-08 |
| Socs1 | 1.031516671 | 0.000211 |
| Krt19 | 1.031899557 | 0.003242 |
| Gchfr | 1.038505393 | 0.001442 |
| Rnase13 | 1.039887351 | 0.001001 |
| Aldob | 1.044959864 | 8.57E-05 |
| Adrb1 | 1.049589941 | 0.008705 |
| Acot4 | 1.055625373 | 0.000978 |
| Dnajb13 | 1.058458947 | 4.84E-10 |
| Serpinf2 | 1.064527445 | 0.002738 |
| 1700056E22Rik | 1.066491967 | 0.005286 |
| Rasal1 | 1.078930281 | 4.31E-06 |
| Dusp4 | 1.079576047 | 9.59E-08 |
| Tmem82 | 1.093079365 | 0.006987 |
| Cd5 | 1.094510179 | 4.94E-06 |
| Ihh | 1.09643528 | 4.63E-06 |
| Rrad | 1.102893359 | 6.98E-11 |
| Kif5a | 1.109697368 | 0.032339 |
| Batf3 | 1.130658348 | 2.85E-05 |
| Cd300e | 1.14640238 | 1.49E-06 |
| Wfikkn1 | 1.1499094 | 0.004738 |
| Fgfbp3 | 1.155550487 | 0.019173 |
| Oasl1 | 1.159191951 | 3.17E-09 |
| Chkb | 1.184612675 | 0.001881 |
| Tppp2 | 1.187824513 | 0.002757 |
| Hbq1a | 1.21282657 | 2.73E-10 |
| Lmtk3 | 1.214414475 | 0.008085 |
| Isg15 | 1.258086229 | 1.60E-14 |
| Cecr6 | 1.258511039 | 0.000579 |
| Tmem221 | 1.264314475 | 1.55E-05 |
| Irf7 | 1.27465516 | 4.40E-16 |
| Derl3 | 1.280305777 | 0.000156 |
| Rab15 | 1.325520327 | 1.84E-08 |
| Apol9a | 1.337121789 | 5.30E-05 |
| Tmem121 | 1.361962855 | 0.003387 |
| Acot1 | 1.419660414 | 1.43E-12 |
| Odf3b | 1.423443506 | 0.000341 |
| Cyp1a1 | 1.441895633 | 4.61E-08 |
| Acot5 | 1.467689621 | 6.26E-07 |
| Ffar1 | 1.476778614 | 5.30E-05 |
| Siglec15 | 1.513761985 | 9.13E-09 |
| Il9r | 1.534887734 | 4.06E-08 |
| Sgk2 | 1.539266506 | 0.000335 |
| Nyap2 | 1.541351273 | 0.000186 |
| Flywch2 | 1.620925898 | 1.23E-05 |
| Slc1a2 | 1.639015179 | 0.042068 |
| Acot3 | 1.722575423 | 4.19E-07 |
| Gdnf | 1.77245226 | 8.20E-06 |
| Chrna2 | 1.829280224 | 1.21E-17 |
| Ttr | 1.954698868 | 0.006239 |
| Cytl1* | 0.251 | 0.133164 |
|  |  |  |

***not significantly changed**

**Supplementary Table 2.** Bulk RNA seq data from control joints compared to type 1 diabetes mellitus joints. Fold downregulation (log2 scale).

| **Gene** | **Expression** | **Adjusted p-Value** |
| --- | --- | --- |
| Lep | -3.475814884 | 1.21E-11 |
| Lrrc52 | -1.986646701 | 8.46E-18 |
| Ostn | -1.858750812 | 3.66E-08 |
| Nr4a3 | -1.831968361 | 9.44E-22 |
| Trim67 | -1.822835446 | 2.95E-07 |
| Mmp12 | -1.736728613 | 1.21E-07 |
| Sycp3 | -1.687993953 | 3.76E-08 |
| Cdsn | -1.629926117 | 7.97E-05 |
| Mettl21e | -1.580274787 | 1.48E-08 |
| Frmd7 | -1.544912175 | 1.68E-10 |
| Sucnr1 | -1.541556178 | 0.000255444 |
| Ddit4 | -1.522470551 | 8.51E-07 |
| Slc25a25 | -1.502366948 | 2.63E-14 |
| Zfp758 | -1.495665266 | 3.29E-16 |
| Prrg1 | -1.476839452 | 5.03E-08 |
| 4933415F23Rik | -1.440525905 | 9.22E-06 |
| Tecta | -1.4188325 | 1.22E-05 |
| Klhdc7a | -1.411975946 | 1.23E-09 |
| Mrgpra9 | -1.400582316 | 6.05E-05 |
| Zfp943 | -1.377070049 | 8.46E-18 |
| Ccdc39 | -1.345458561 | 2.36E-07 |
| Cnksr2 | -1.343833462 | 3.38E-06 |
| Tceal7 | -1.343725572 | 3.79E-07 |
| Slc10a6 | -1.342470231 | 1.68E-10 |
| Arrdc2 | -1.316666211 | 2.92E-09 |
| Spata9 | -1.30151081 | 0.001220334 |
| Cdh4 | -1.296789534 | 1.76E-07 |
| Zfp946 | -1.292658511 | 2.91E-12 |
| Gucy1a2 | -1.281191896 | 1.79E-10 |
| Esrrg | -1.279386671 | 6.22E-16 |
| Nmrk2 | -1.27746539 | 2.63E-08 |
| Zfp942 | -1.27691336 | 3.93E-12 |
| Aqp4 | -1.276034893 | 5.17E-08 |
| Ednrb | -1.264077414 | 2.69E-17 |
| Nnat | -1.254026905 | 1.59E-11 |
| Bdh1 | -1.245575594 | 3.66E-13 |
| Slc4a10 | -1.240359986 | 6.09E-07 |
| Grb14 | -1.239278944 | 1.74E-18 |
| F830016B08Rik | -1.236210989 | 5.87E-13 |
| Gulp1 | -1.229964923 | 4.70E-07 |
| Tph1 | -1.208900259 | 0.000256318 |
| Fam107a | -1.205604105 | 1.29E-06 |
| Zfp994 | -1.199286507 | 2.02E-16 |
| Ppara | -1.18217426 | 1.03E-08 |
| Hoxd10 | -1.161631159 | 7.09E-06 |
| Raver2 | -1.160686911 | 3.54E-09 |
| Dach2 | -1.160589424 | 4.06E-08 |
| Aspn | -1.149815592 | 1.48E-09 |
| Clec2e | -1.148775528 | 0.000365512 |
| Nat1 | -1.148728776 | 0.000770914 |
| Fgf10 | -1.147486528 | 0.000810994 |
| Ccdc122 | -1.14004293 | 7.94E-06 |
| Cidec | -1.13911578 | 1.10E-05 |
| D430041D05Rik | -1.126701152 | 2.59E-11 |
| Zfp947 | -1.117898737 | 1.36E-07 |
| Olfr78 | -1.11048579 | 4.03E-10 |
| Oxct1 | -1.106339688 | 2.12E-17 |
| Npy1r | -1.09830659 | 4.75E-10 |
| Zfp52 | -1.097755795 | 2.99E-13 |
| Stc1 | -1.088897859 | 4.72E-05 |
| Sh3bgrl | -1.083910474 | 5.65E-09 |
| Eqtn | -1.082493229 | 0.001564013 |
| Ppp1r3a | -1.080420597 | 3.39E-12 |
| Per1 | -1.0745532 | 3.62E-07 |
| Fhl1 | -1.070187066 | 3.08E-15 |
| Carnmt1 | -1.069449832 | 1.85E-15 |
| Mybph | -1.068891254 | 0.006988778 |
| Gdap1 | -1.063088226 | 8.27E-14 |
| Zfp975 | -1.062801767 | 3.43E-09 |
| Slf1 | -1.062041932 | 2.72E-15 |
| Zfp944 | -1.055210978 | 6.95E-08 |
| Nr4a2 | -1.046510191 | 0.004597155 |
| Ugp2 | -1.040229926 | 3.32E-20 |
| Saa3 | -1.039761808 | 0.015379623 |
| Pkdrej | -1.033594767 | 0.001210648 |
| Kcnab1 | -1.032902932 | 6.13E-09 |
| Hecw2 | -1.031904157 | 4.21E-10 |
| Pfn4 | -1.030262977 | 0.009947962 |
| Npr3 | -1.027586716 | 7.50E-06 |
| Apold1 | -1.023500617 | 9.50E-06 |
| Lrp2bp | -1.022871224 | 8.43E-08 |
| Gm28557 | -1.01546953 | 3.07E-08 |
| Dusp18 | -1.014315289 | 6.51E-15 |
| Bche | -1.006033702 | 2.48E-09 |
| Mpped2 | -1.001651936 | 3.60E-08 |
| Aspa | -1.000910403 | 4.02E-08 |
| Gck | -0.996319925 | 6.14E-06 |
| Lypd6 | -0.996286894 | 4.49E-05 |
| Atp1b1 | -0.994854732 | 1.65E-13 |
| Cops2 | -0.99309044 | 1.60E-14 |
| Cav2 | -0.992817645 | 1.85E-12 |
| Pi15 | -0.992612406 | 1.46E-05 |
| Elovl4 | -0.992513732 | 0.022733236 |
| Trpm1 | -0.992265146 | 0.00131227 |
| Pld5 | -0.990454127 | 3.23E-06 |
| Tmem132b | -0.989045338 | 1.94E-05 |
| Btc | -0.983786909 | 0.000299491 |
| Abcb7 | -0.98029591 | 1.43E-12 |
| Atp11c | -0.976372304 | 8.15E-09 |
| Gm4841 | -0.974734771 | 0.000101162 |
| Chm | -0.974272391 | 2.92E-09 |
| Arrdc3 | -0.972899062 | 2.66E-17 |
| Ppp1r3c | -0.96987451 | 1.60E-13 |
| 8430408G22Rik | -0.965369207 | 3.29E-09 |
| Fosb | -0.96534711 | 0.037317413 |
| Slc15a5 | -0.965156541 | 0.00224573 |
| Scd1 | -0.963532054 | 6.40E-05 |
| Ptpn3 | -0.962685098 | 1.02E-09 |
| Fsd1l | -0.958781542 | 7.12E-12 |
| Cd28 | -0.957876937 | 5.80E-05 |
| Adgrf4 | -0.956558457 | 0.003328997 |
| Smarca1 | -0.95547599 | 3.77E-11 |
| Cfap43 | -0.951791469 | 0.000821185 |
| Clec4e | -0.950984286 | 2.68E-05 |
| Sim1 | -0.947111369 | 8.57E-05 |
| Tsc22d3 | -0.94639516 | 8.35E-16 |
| D3Ertd751e | -0.944799295 | 8.02E-09 |
| Coq10b | -0.941349546 | 3.18E-13 |
| Zfp612 | -0.940646595 | 9.27E-11 |
| Fam184b | -0.93975503 | 0.013281767 |
| Tob1 | -0.938202393 | 4.53E-09 |
| Neto2 | -0.937285339 | 6.42E-07 |
| Sgk1 | -0.935242013 | 8.66E-15 |
| Itgb3bp | -0.934172678 | 5.65E-08 |
| Cacna2d4 | -0.933589951 | 5.30E-05 |
| Tfcp2l1 | -0.9275188 | 1.16E-08 |
| Dupd1 | -0.927022961 | 1.78E-08 |
| 1810022K09Rik | -0.924638242 | 6.29E-12 |
| Ppargc1a | -0.921177023 | 2.51E-10 |
| Tspan12 | -0.920111501 | 2.47E-12 |
| Glb1l2 | -0.91861969 | 1.70E-05 |
| Hs3st5 | -0.917337225 | 2.23E-07 |
| Ccdc85a | -0.91722778 | 2.17E-10 |
| Ube2d3 | -0.911625312 | 2.14E-15 |
| Rnf128 | -0.909790714 | 3.88E-12 |
| Ctnna3 | -0.909132866 | 5.94E-10 |
| Kcna5 | -0.907635453 | 2.95E-08 |
| Yes1 | -0.904429171 | 9.57E-08 |
| Dnajb9 | -0.901988121 | 3.40E-08 |
| Map2k6 | -0.901246074 | 9.40E-09 |
| Lpl | -0.900417566 | 1.74E-18 |
| Cbr2 | -0.898953372 | 2.65E-08 |
| Pck1 | -0.897646066 | 1.39E-05 |
| Mpzl2 | -0.897392374 | 0.010611309 |
| Ube2g1 | -0.896644406 | 4.84E-10 |
| Asxl3 | -0.896634942 | 0.020967443 |
| Lgals12 | -0.895104602 | 1.72E-09 |
| Prune2 | -0.890167131 | 2.56E-07 |
| Tmed5 | -0.88820962 | 3.14E-07 |
| Grem2 | -0.887732708 | 0.000820229 |
| Dgkb | -0.887468338 | 0.000605439 |
| Bcap29 | -0.886481699 | 3.10E-10 |
| Arhgap5 | -0.883795582 | 4.80E-13 |
| Qk | -0.879775422 | 2.16E-08 |
| Omd | -0.879592911 | 0.000761096 |
| Fam126b | -0.878523275 | 1.62E-06 |
| Fam199x | -0.87714822 | 1.01E-05 |
| Lrif1 | -0.875227761 | 2.36E-09 |
| Cep126 | -0.872769577 | 0.000501367 |
| Dnm1l | -0.871639972 | 2.68E-14 |
| Nr4a1 | -0.871281337 | 2.64E-08 |
| Zfp459 | -0.871142899 | 0.004676435 |
| Gm5141 | -0.867385228 | 0.002022689 |
| Thpo | -0.866188008 | 8.28E-09 |
| Cfl2 | -0.864928364 | 3.66E-13 |
| Pln | -0.86461577 | 0.000173015 |
| Mstn | -0.864248979 | 7.42E-09 |
| Chpt1 | -0.863970701 | 6.79E-17 |
| Mfsd7b | -0.863579961 | 0.000461939 |
| Tmem88b | -0.863223167 | 0.000397956 |
| Mmachc | -0.862606451 | 3.39E-12 |
| Lyrm7 | -0.862515495 | 1.18E-08 |
| Lyrm1 | -0.860755838 | 3.79E-07 |
| Map9 | -0.858698879 | 1.26E-05 |
| Rcan2 | -0.857532452 | 2.84E-10 |
| Zyg11a | -0.857278753 | 0.001005979 |
| Pon1 | -0.8571108 | 0.000695572 |
| Adora1 | -0.850897645 | 0.037197266 |
| Glrb | -0.848764034 | 0.032864856 |
| Snx16 | -0.848639201 | 8.57E-07 |
| Rgs1 | -0.845001544 | 0.001437936 |
| Mob4 | -0.843448676 | 1.03E-12 |
| Serpina3c | -0.838282183 | 0.000511148 |
| Fgf13 | -0.837187416 | 7.90E-10 |
| Hspb6 | -0.836985108 | 8.62E-09 |
| Atg10 | -0.835496723 | 1.97E-10 |
| Zbtb10 | -0.835484419 | 2.52E-08 |
| Stbd1 | -0.833517232 | 1.34E-09 |
| Tmem263 | -0.833476149 | 4.29E-07 |
| Apoo | -0.833187061 | 4.56E-10 |
| Lnpk1 | -0.832545593 | 4.77E-09 |
| Rnpc3 | -0.831530572 | 4.25E-07 |
| B3galt1 | -0.829646837 | 6.15E-07 |
| Cyp2e1 | -0.82964589 | 0.001349136 |
| Rnf138 | -0.827935944 | 2.52E-08 |
| Fgfbp1 | -0.826967901 | 0.00344466 |
| Pm20d1 | -0.825512724 | 0.009815089 |
| Ube2v2 | -0.825048414 | 8.57E-15 |
| Chordc1 | -0.823294991 | 8.62E-09 |
| Zfp120 | -0.822851409 | 2.02E-10 |
| Mbnl2 | -0.822172437 | 7.91E-07 |
| Plagl1 | -0.821472478 | 0.000690971 |
| Iigp1 | -0.820080681 | 5.45E-05 |
| Mef2c | -0.819920007 | 1.23E-11 |
| Lypla1 | -0.819888145 | 8.95E-11 |
| Nrep | -0.819728235 | 1.16E-05 |
| Tmx3 | -0.817565013 | 6.20E-06 |
| Nek5 | -0.816982794 | 0.010348959 |
| Cntnap2 | -0.816476628 | 0.011857048 |
| Ccdc38 | -0.815898457 | 0.020972044 |
| Fos | -0.815723339 | 4.91E-05 |
| Fhl5 | -0.815148083 | 0.002909305 |
| Rrm2b | -0.812046003 | 1.36E-09 |
| Mpc1 | -0.81097953 | 3.49E-11 |
| Fmr1 | -0.810397358 | 6.86E-07 |
| Kcnq5 | -0.810291635 | 1.51E-05 |
| Ube3a | -0.809537886 | 3.44E-12 |
| Zfp977 | -0.808539938 | 2.44E-06 |
| Hk2 | -0.807878647 | 5.79E-10 |
| Trp53inp1 | -0.807809957 | 2.03E-06 |
| Ccnc | -0.807357313 | 4.41E-05 |
| Snhg11 | -0.806621106 | 3.59E-05 |
| Fam19a3 | -0.806496717 | 3.58E-07 |
| Bmi1 | -0.80586127 | 9.27E-08 |
| Kitl | -0.805472373 | 9.59E-08 |
| Sbk3 | -0.805221114 | 0.00382575 |
| Gpr137c | -0.803930907 | 0.000164721 |
| Slc4a4 | -0.803454993 | 4.43E-08 |
| Nxt2 | -0.802669813 | 3.73E-05 |
| Best3 | -0.802662746 | 2.19E-10 |
| Lonrf2 | -0.800072568 | 0.005160033 |
| Bmpr1b | -0.794167364 | 1.87E-07 |
| Car8 | -0.794087333 | 2.72E-08 |
| Timp4 | -0.793971716 | 0.00034111 |
| Zfp51 | -0.793086919 | 3.13E-08 |
| Kcnn2 | -0.793018595 | 0.002272287 |
| Chml | -0.792755648 | 7.37E-05 |
| Zfp948 | -0.7913823 | 1.23E-06 |
| 1810011O10Rik | -0.79027924 | 2.83E-07 |
| Slc35f1 | -0.790237505 | 0.006024126 |
| Lyrm5 | -0.789426875 | 2.91E-12 |
| Krt222 | -0.787146502 | 0.00090908 |
| Cuzd1 | -0.786379553 | 0.006820891 |
| Kera | -0.784762881 | 6.92E-07 |
| Tiparp | -0.784120859 | 8.52E-11 |
| Dok5 | -0.783837793 | 3.24E-05 |
| Dld | -0.7835017 | 8.31E-13 |
| Zfp518a | -0.782740069 | 8.86E-06 |
| Zfp386 | -0.781306666 | 1.28E-08 |
| Dram2 | -0.78046128 | 6.66E-10 |
| Homer1 | -0.778650276 | 4.34E-08 |
| A830080D01Rik | -0.778579586 | 1.54E-05 |
| Tmem47 | -0.777736642 | 9.77E-05 |
| Ythdf3 | -0.777314162 | 4.70E-07 |
| Trove2 | -0.777216085 | 1.53E-06 |
| Cacna2d1 | -0.776010838 | 1.06E-09 |
| Tigd4 | -0.775343541 | 8.65E-10 |
| Naa50 | -0.774350028 | 3.57E-11 |
| Cd164 | -0.773203023 | 3.56E-16 |
| Pde7a | -0.772466082 | 2.31E-11 |
| Perp | -0.772326701 | 0.000348786 |
| Lcorl | -0.771594314 | 1.81E-06 |
| Taf9b | -0.77112392 | 1.65E-06 |
| Zfp770 | -0.770728766 | 8.65E-10 |
| Fcho2 | -0.769447272 | 1.50E-05 |
| Pcmtd1 | -0.768155966 | 5.10E-09 |
| Otud1 | -0.767076317 | 0.000101448 |
| Zfp558 | -0.766101504 | 0.000758581 |
| Serinc1 | -0.766019849 | 6.53E-08 |
| Cycs | -0.765344261 | 2.95E-11 |
| Cnga3 | -0.764776481 | 0.002320344 |
| Lysmd3 | -0.764663113 | 7.56E-05 |
| Epb41l5 | -0.764604137 | 4.01E-05 |
| Micu3 | -0.764069073 | 1.38E-05 |
| Ckmt2 | -0.763627333 | 5.96E-09 |
| Tmem14a | -0.763604124 | 0.000477578 |
| Gnai1 | -0.762958356 | 8.66E-09 |
| Prss23 | -0.762884969 | 1.87E-09 |
| Tnfaip6 | -0.762646319 | 0.000472595 |
| Ppp1cb | -0.762494351 | 1.01E-10 |
| C1d | -0.762392197 | 1.26E-08 |
| Yy2 | -0.761215505 | 0.001060517 |
| Ogn | -0.759094874 | 3.27E-09 |
| Tmem266 | -0.758541201 | 0.001228339 |
| Usp2 | -0.758508527 | 2.05E-09 |
| Fkbp3 | -0.756960801 | 5.71E-07 |
| 2010315B03Rik | -0.755590059 | 1.03E-07 |
| Ptbp2 | -0.755086582 | 5.71E-07 |
| Hccs | -0.754718697 | 3.29E-09 |
| Rpp40 | -0.754613718 | 6.43E-07 |
| Igf1 | -0.754557968 | 8.08E-07 |
| Spaca1 | -0.754275731 | 0.039231718 |
| Prpf39 | -0.754174033 | 2.09E-06 |
| Fancd2os | -0.753029064 | 0.048475345 |
| Ldhb | -0.752369683 | 1.85E-07 |
| Mterf2 | -0.749000995 | 4.58E-10 |
| Mzt1 | -0.74738006 | 3.33E-11 |
| Kras | -0.747165875 | 4.40E-07 |
| 7-Sep | -0.746747467 | 1.55E-06 |
| Nufip2 | -0.746629358 | 2.98E-08 |
| Idh3a | -0.746403534 | 4.70E-10 |
| Cetn4 | -0.745667767 | 0.034392659 |
| Ccdc50 | -0.745066844 | 2.05E-09 |
| Cryab | -0.744638326 | 1.08E-08 |
| Cyp2c44 | -0.741120761 | 0.000838043 |
| Rc3h2 | -0.739411253 | 2.02E-10 |
| Far1 | -0.739212206 | 3.84E-05 |
| Cabyr | -0.739052995 | 0.041950442 |
| 9330159F19Rik | -0.73892166 | 1.31E-09 |
| Rab10 | -0.737902412 | 1.65E-11 |
| Cul3 | -0.73772851 | 1.29E-09 |
| Ucn2 | -0.737514948 | 0.005553834 |
| Lnx1 | -0.737251943 | 2.72E-06 |
| Cbfb | -0.736795825 | 5.65E-08 |
| Acadsb | -0.735094073 | 1.35E-09 |
| Slc30a2 | -0.733982649 | 0.000398912 |
| P2ry10 | -0.733982339 | 0.009670959 |
| Palmd | -0.733865518 | 8.92E-08 |
| Perm1 | -0.732497384 | 2.92E-08 |
| Nr1d2 | -0.732326842 | 1.50E-09 |
| Plcd4 | -0.731897605 | 1.14E-07 |
| Mmgt1 | -0.731702018 | 2.97E-09 |
| Myh1 | -0.731428315 | 8.95E-11 |
| Npnt | -0.73141298 | 2.17E-05 |
| Slc47a1 | -0.730758392 | 1.27E-07 |
| Enpp4 | -0.730515296 | 2.20E-13 |
| Prkaa2 | -0.730323528 | 1.84E-09 |
| Ugt8a | -0.728599419 | 0.008824284 |
| Xrra1 | -0.728192758 | 0.039599287 |
| Ccng1 | -0.728057563 | 1.95E-07 |
| Smim10l1 | -0.728030958 | 6.71E-06 |
| Ndufaf4 | -0.72677141 | 6.77E-09 |
| Lin7a | -0.725966726 | 0.000242315 |
| Errfi1 | -0.725486763 | 1.98E-05 |
| Slc25a40 | -0.724501198 | 3.17E-06 |
| Nbeal1 | -0.724261256 | 7.46E-08 |
| Ddo | -0.723938626 | 7.98E-11 |
| 4921536K21Rik | -0.72335769 | 0.0355672 |
| Ttc19 | -0.723131806 | 1.01E-11 |
| Zfp759 | -0.722913196 | 4.94E-05 |
| Kpna3 | -0.722591332 | 8.56E-11 |
| Adra1a | -0.722478195 | 0.003403787 |
| Smco1 | -0.722302643 | 1.26E-05 |
| Gbe1 | -0.719640447 | 1.16E-08 |
| Cdkl2 | -0.719357805 | 7.00E-06 |
| Themis3 | -0.718986286 | 0.024671535 |
| Mettl21c | -0.718611641 | 6.48E-05 |
| Rap2c | -0.718398854 | 2.86E-09 |
| Kpna4 | -0.716254284 | 4.87E-11 |
| Orc4 | -0.716252523 | 2.18E-09 |
| Hook1 | -0.715532481 | 5.26E-08 |
| Dpt | -0.715332979 | 6.13E-09 |
| Tmem68 | -0.714917187 | 6.29E-06 |
| Foxo6 | -0.71447062 | 0.006385079 |
| Tmem106b | -0.714058274 | 6.06E-08 |
| Esr1 | -0.713944192 | 2.91E-08 |
| Dynlt3 | -0.713624402 | 7.15E-09 |
| Zfp930 | -0.7134127 | 2.41E-07 |
| Tmem65 | -0.713012295 | 3.50E-10 |
| Myot | -0.712784227 | 4.40E-07 |
| Spata1 | -0.71226206 | 6.66E-05 |
| Gsta4 | -0.712039755 | 5.81E-06 |
| Ghr | -0.711576433 | 2.92E-09 |
| Scrn3 | -0.711205124 | 4.00E-09 |
| Bbs5 | -0.710923965 | 3.78E-05 |
| Vbp1 | -0.709281719 | 7.10E-10 |
| Hnmt | -0.708707939 | 2.61E-05 |
| Cd55b | -0.707879007 | 3.65E-06 |
| Rhobtb3 | -0.707765264 | 1.07E-08 |
| Rnft1 | -0.707349241 | 3.61E-10 |
| Gpr34 | -0.706569246 | 0.001785021 |
| Ankrd29 | -0.705836699 | 0.000144594 |
| Ccdc73 | -0.705729477 | 8.85E-05 |
| Eya1 | -0.705105852 | 3.61E-08 |
| Stau2 | -0.701636269 | 7.03E-09 |
| Cpeb2 | -0.70129708 | 4.29E-07 |
| Lmcd1 | -0.701205876 | 1.52E-06 |
| Ppp1r42 | -0.701122476 | 0.000433466 |
| Mblac2 | -0.7010102 | 0.000430473 |
| Bbof1 | -0.7003742 | 0.001349136 |
| Dzip3 | -0.699696696 | 0.000124297 |
| Rab18 | -0.699255493 | 1.14E-08 |
| Cul5 | -0.699228629 | 4.48E-08 |
| Frk | -0.698718319 | 0.002251304 |
| Nos1 | -0.698262213 | 8.86E-08 |
| Pja2 | -0.698117392 | 4.97E-10 |
| Ogdhl | -0.69801376 | 0.01295327 |
| Nlrp10 | -0.697649825 | 0.006895467 |
| Robo2 | -0.69723031 | 0.018651522 |
| Actr3b | -0.696511204 | 0.000669824 |
| Lancl3 | -0.695206866 | 0.030390378 |
| Klf9 | -0.694819775 | 1.25E-07 |
| Mpdz | -0.69464089 | 5.45E-10 |
| Zfp40 | -0.694049804 | 0.000314147 |
| Il33 | -0.693884304 | 0.001174796 |
| Morn4 | -0.693440186 | 0.000395349 |
| Phip | -0.692211112 | 0.000121872 |
| Zfp72 | -0.69183006 | 0.002283366 |
| Atad1 | -0.690285342 | 2.28E-10 |
| Myh3 | -0.689219237 | 6.12E-07 |
| Zfp729b | -0.689200774 | 7.09E-06 |
| 4930486L24Rik | -0.688908035 | 0.015382241 |
| Manea | -0.688494023 | 1.56E-05 |
| Pcdhb18 | -0.688396149 | 0.036284972 |
| Cep85l | -0.685102725 | 9.47E-05 |
| Hs6st2 | -0.68506618 | 0.002990941 |
| Naa30 | -0.684993222 | 4.47E-07 |
| Nexn | -0.6841666 | 9.72E-05 |
| Frzb | -0.683820662 | 6.27E-05 |
| Lin7c | -0.683717211 | 5.02E-05 |
| Ids | -0.68350797 | 5.33E-07 |
| Asb15 | -0.681344235 | 4.77E-06 |
| Slc1a1 | -0.681340926 | 2.37E-05 |
| Pdlim5 | -0.681062284 | 2.50E-08 |
| Zfp677 | -0.680653845 | 0.00015758 |
| Lrch2 | -0.680621817 | 0.023522928 |
| Sik1 | -0.679925868 | 0.00039869 |
| Cacybp | -0.676582161 | 3.80E-10 |
| Nox4 | -0.676129393 | 0.001354224 |
| Zfp53 | -0.675484581 | 0.000185671 |
| Tmc7 | -0.675452764 | 3.97E-06 |
| Dbt | -0.675311106 | 3.18E-08 |
| D430019H16Rik | -0.674793644 | 0.027216589 |
| 3830406C13Rik | -0.673839809 | 1.90E-08 |
| Mut | -0.673300437 | 1.22E-07 |
| Ppp4r4 | -0.673290539 | 0.01082288 |
| St8sia5 | -0.673107985 | 2.72E-07 |
| Il1r2 | -0.672864027 | 0.00082518 |
| Myo5b | -0.671841858 | 0.015688732 |
| Kbtbd3 | -0.670734894 | 2.31E-05 |
| Fnip1 | -0.670109058 | 2.58E-07 |
| Zfp54 | -0.669923536 | 0.000725303 |
| Dcun1d1 | -0.669437692 | 5.46E-06 |
| 1810041L15Rik | -0.668769024 | 0.016192413 |
| AW549877 | -0.668709701 | 1.99E-07 |
| Lactb2 | -0.668496175 | 1.53E-08 |
| Mb | -0.668494266 | 0.000419041 |
| Dmd | -0.668155559 | 5.03E-08 |
| Abhd18 | -0.668042207 | 2.60E-05 |
| Ptprr | -0.667836733 | 0.003433664 |
| Atp5s | -0.667633895 | 7.33E-08 |
| Hoxd8 | -0.665394247 | 0.001521818 |
| Hsbp1l1 | -0.665091343 | 0.00723625 |
| Dach1 | -0.664712748 | 0.001812835 |
| Ppp1r14c | -0.662146663 | 3.56E-05 |
| Rasl2-9 | -0.66161703 | 0.018579519 |
| Mbnl1 | -0.661578144 | 0.000324285 |
| Tmem69 | -0.660786405 | 9.59E-08 |
| Ptprg | -0.660699617 | 1.64E-09 |
| Tmem30a | -0.660514416 | 1.79E-10 |
| Ikzf2 | -0.660124226 | 8.16E-07 |
| Phf20l1 | -0.659968074 | 3.77E-06 |
| Haus3 | -0.659088026 | 0.000166754 |
| Lmod3 | -0.658702569 | 7.34E-06 |
| Camta1 | -0.658461057 | 7.09E-06 |
| Tcaim | -0.658415006 | 1.70E-06 |
| Trim23 | -0.657491537 | 6.10E-09 |
| Slc25a46 | -0.657294952 | 7.14E-08 |
| Ppp3cb | -0.656758023 | 1.01E-10 |
| Sema3e | -0.656347348 | 0.003292611 |
| Scn7a | -0.656266224 | 8.92E-06 |
| Ube2w | -0.655183061 | 6.51E-06 |
| Fndc5 | -0.654046304 | 1.21E-06 |
| Cab39 | -0.653840744 | 1.94E-10 |
| Larp4 | -0.653467283 | 1.13E-05 |
| Thrsp | -0.653437295 | 1.89E-08 |
| Acvr1c | -0.653294376 | 0.005805149 |
| Fam228b | -0.65279056 | 0.035097721 |
| Esrrb | -0.651885923 | 0.000359491 |
| Blzf1 | -0.651796316 | 5.24E-06 |
| Smpx | -0.651211512 | 3.11E-08 |
| Rnf11 | -0.65028258 | 3.53E-09 |
| Muc1 | -0.650225415 | 0.009443511 |
| Prkg1 | -0.65012339 | 9.87E-06 |
| Syap1 | -0.649888622 | 2.43E-06 |
| Tob2 | -0.649871371 | 5.33E-07 |
| Strn3 | -0.649689912 | 1.48E-08 |
| Rnf2 | -0.649535464 | 9.30E-05 |
| Acsl4 | -0.64936591 | 8.84E-09 |
| Acbd5 | -0.649332786 | 2.60E-05 |
| Kansl1l | -0.648232067 | 5.39E-06 |
| Pcdhb14 | -0.648009727 | 0.014327975 |
| Gldn | -0.647964928 | 0.01434713 |
| Gnpnat1 | -0.6476755 | 3.40E-07 |
| Tmem45b | -0.647385323 | 0.024320856 |
| Samd8 | -0.646890395 | 1.01E-09 |
| Cdnf | -0.646600791 | 1.00E-06 |
| Slc2a4 | -0.646241897 | 4.77E-05 |
| Trdn | -0.646003105 | 2.83E-06 |
| Smim8 | -0.64542082 | 8.19E-07 |
| Chac2 | -0.645153456 | 1.07E-06 |
| Nampt | -0.644111466 | 4.27E-08 |
| Pkia | -0.643917818 | 2.28E-08 |
| Cpsf6 | -0.643433718 | 4.70E-05 |
| 2610008E11Rik | -0.642893249 | 1.23E-06 |
| Prrx1 | -0.641857339 | 5.67E-06 |
| Xirp2 | -0.641470121 | 1.97E-07 |
| Tfb2m | -0.640847821 | 8.28E-09 |
| Sec24a | -0.640769974 | 7.87E-05 |
| Map3k6 | -0.640327717 | 8.21E-05 |
| Col4a3 | -0.640028318 | 0.005806238 |
| Wwp1 | -0.639218468 | 1.47E-06 |
| Hsph1 | -0.639063053 | 0.000112986 |
| Pon3 | -0.638663216 | 5.78E-07 |
| Sypl | -0.638540537 | 3.91E-08 |
| Uchl3 | -0.638183646 | 1.94E-05 |
| Thrb | -0.637946774 | 1.13E-05 |
| Cbll1 | -0.637702993 | 4.70E-06 |
| Zbtb6 | -0.637653778 | 0.002228068 |
| Abca1 | -0.636384221 | 0.00015779 |
| Nme7 | -0.636292649 | 3.41E-05 |
| 9430076C15Rik | -0.635620676 | 0.011599604 |
| Ptbp3 | -0.63471176 | 0.000624443 |
| Tead1 | -0.6340263 | 2.04E-07 |
| Tceal5 | -0.633428738 | 0.000205931 |
| Yme1l1 | -0.633284545 | 3.18E-08 |
| Fastkd1 | -0.633091053 | 8.92E-08 |
| Naa15 | -0.633070862 | 4.90E-08 |
| S1pr1 | -0.632893555 | 2.55E-09 |
| Ube2q2 | -0.632768058 | 8.73E-09 |
| Ube2e1 | -0.632423795 | 5.95E-06 |
| Tbc1d19 | -0.632087713 | 5.92E-07 |
| Rb1cc1 | -0.63191415 | 3.79E-08 |
| 2610002M06Rik | -0.631913162 | 3.40E-07 |
| Cnep1r1 | -0.631507863 | 8.86E-08 |
| Mitf | -0.630633505 | 1.23E-06 |
| Amot | -0.630492677 | 3.54E-08 |
| Arsk | -0.629481569 | 7.29E-05 |
| Rab1a | -0.6293658 | 9.84E-09 |
| Ano5 | -0.628314743 | 1.29E-09 |
| Mob1b | -0.628058835 | 5.96E-07 |
| Pcm1 | -0.627639914 | 1.45E-06 |
| Otog | -0.627414311 | 0.044509121 |
| Taok1 | -0.627114407 | 5.65E-08 |
| Slc25a36 | -0.627085384 | 5.86E-05 |
| Trp63 | -0.626852862 | 3.29E-06 |
| Medag | -0.626331191 | 3.16E-06 |
| Rnf13 | -0.62615751 | 7.14E-08 |
| Exoc5 | -0.625095816 | 1.18E-07 |
| Zdhhc2 | -0.625080099 | 0.014923692 |
| Fyttd1 | -0.624413364 | 1.22E-11 |
| Acvr2a | -0.624362441 | 2.19E-06 |
| Dmxl1 | -0.624244412 | 1.97E-06 |
| Ppid | -0.624126229 | 1.48E-06 |
| Gmfb | -0.623996535 | 7.47E-08 |
| Mybpc1 | -0.623336427 | 4.44E-05 |
| Rgs7bp | -0.623317117 | 0.009932175 |
| Sp4 | -0.623214082 | 0.000456041 |
| Zfp709 | -0.621994158 | 0.000258451 |
| Tmem37 | -0.621441727 | 0.027579017 |
| E130311K13Rik | -0.621111093 | 0.000338719 |
| Actr6 | -0.62090283 | 7.93E-06 |
| Gnpda2 | -0.620654865 | 0.00026572 |
| Klf6 | -0.620634543 | 2.83E-06 |
| Uba6 | -0.620595497 | 2.18E-05 |
| 6720489N17Rik | -0.61977064 | 0.006278624 |
| Cntfr | -0.619345717 | 0.00196658 |
| Bmt2 | -0.619260425 | 1.67E-06 |
| Zfp65 | -0.619001156 | 1.89E-05 |
| Kif21a | -0.61869455 | 9.89E-07 |
| Hook3 | -0.618479605 | 1.11E-06 |
| Pfkfb1 | -0.618088186 | 1.51E-05 |
| Cav1 | -0.617959459 | 3.69E-06 |
| Mpp5 | -0.617901504 | 2.34E-08 |
| Tmed7 | -0.617671051 | 7.89E-05 |
| Zfand6 | -0.617627894 | 1.79E-08 |
| Kctd12b | -0.617459328 | 9.72E-05 |
| Zfp455 | -0.617270285 | 0.014545049 |
| Calcrl | -0.617197419 | 1.40E-05 |
| Pptc7 | -0.617099254 | 3.99E-10 |
| Cd200r4 | -0.616966289 | 0.018380051 |
| Gpbp1 | -0.616593323 | 4.29E-07 |
| Phf6 | -0.616377685 | 3.45E-05 |
| Foxp2 | -0.616133705 | 0.004194753 |
| Fign | -0.616130576 | 0.001467041 |
| Lztfl1 | -0.615768123 | 4.77E-05 |
| Pnpla8 | -0.615721558 | 4.04E-09 |
| Itgb6 | -0.615682627 | 4.94E-05 |
| Chl1 | -0.61467216 | 0.005503131 |
| Scamp1 | -0.614500423 | 9.40E-09 |
| Ccdc173 | -0.61437578 | 0.028766796 |
| Pgbd1 | -0.612413579 | 0.013717912 |
| Cnot7 | -0.612170392 | 6.59E-06 |
| Clec4d | -0.611771406 | 0.000136784 |
| Sgms1 | -0.611771044 | 1.53E-07 |
| Thoc2 | -0.611290246 | 0.000279982 |
| Them4 | -0.61035703 | 1.54E-05 |
| Atl2 | -0.610182078 | 5.25E-09 |
| Pik3c2a | -0.609524281 | 2.40E-06 |
| Rap1a | -0.609384296 | 8.01E-05 |
| Zfp58 | -0.609138257 | 3.67E-05 |
| Stk3 | -0.609119911 | 3.45E-05 |
| Rab21 | -0.608420408 | 7.06E-07 |
| Pdcd10 | -0.608155154 | 1.63E-05 |
| Hacd1 | -0.607821202 | 0.000991999 |
| Zbtb33 | -0.607416219 | 7.58E-06 |
| Ccdc43 | -0.607406547 | 1.70E-07 |
| Pde4d | -0.607262355 | 1.73E-05 |
| Oxnad1 | -0.606986276 | 7.39E-06 |
| Smyd1 | -0.606884956 | 1.15E-05 |
| Slc2a12 | -0.606873026 | 3.82E-05 |
| 4430402I18Rik | -0.606738718 | 0.012345944 |
| Pno1 | -0.606061613 | 1.30E-08 |
| Rock2 | -0.605716755 | 1.59E-05 |
| Abra | -0.605291451 | 0.002986679 |
| 2310002L09Rik | -0.604273928 | 1.39E-05 |
| Akap2 | -0.604211729 | 1.89E-08 |
| Cept1 | -0.603815806 | 1.30E-09 |
| Cep112 | -0.603450187 | 0.00723625 |
| Cpeb1 | -0.60239928 | 3.35E-09 |
| C7 | -0.602262749 | 0.028791105 |
| Aoc3 | -0.601630037 | 0.000325456 |
| Fbxo11 | -0.600819193 | 3.24E-07 |
| Gng7 | -0.600649663 | 0.002594783 |
| Nr6a1 | -0.600507873 | 0.000230752 |
| C330018D20Rik | -0.600114937 | 4.80E-05 |
| Mtm1 | -0.599951412 | 1.62E-06 |
| Sgtb | -0.599808912 | 0.030276915 |
| Clip4 | -0.599793563 | 2.39E-06 |
| Calcr | -0.599584769 | 0.047406483 |
| Arl8b | -0.599088482 | 4.26E-08 |
| Fam210a | -0.598973336 | 1.62E-08 |
| Cnot4 | -0.598251682 | 6.64E-05 |
| Rsbn1l | -0.598185096 | 0.000915228 |
| Tmem55a | -0.598035901 | 3.53E-08 |
| Tm2d1 | -0.597334891 | 5.50E-06 |
| Ndufaf1 | -0.597217515 | 4.79E-08 |
| Gca | -0.597023469 | 0.000166225 |
| Tmem126a | -0.596903214 | 1.06E-07 |
| Msi2 | -0.596715203 | 5.71E-07 |
| Samsn1 | -0.596703275 | 0.00091925 |
| Ppp2r3a | -0.596211141 | 2.58E-06 |
| Myog | -0.596119913 | 0.002427432 |
| Pde7b | -0.595965986 | 1.13E-07 |
| Tm9sf2 | -0.595790406 | 4.07E-08 |
| Pcmtd2 | -0.595362215 | 2.81E-05 |
| Prex2 | -0.594786117 | 0.001578514 |
| Fam169a | -0.594584113 | 0.03005815 |
| Bckdhb | -0.594417724 | 7.61E-06 |
| Sema3d | -0.593903348 | 0.038628588 |
| Ptp4a2 | -0.593901337 | 5.33E-08 |
| Hibch | -0.593822738 | 1.84E-09 |
| Pex13 | -0.59370788 | 3.63E-07 |
| Ptcd3 | -0.592364853 | 7.12E-09 |
| Dsel | -0.592281426 | 0.006122065 |
| Abcd2 | -0.592061324 | 4.53E-09 |
| 2510009E07Rik | -0.592047921 | 2.47E-06 |
| Kifap3 | -0.592040766 | 8.92E-08 |
| Maf | -0.591249613 | 7.28E-06 |
| Tpp2 | -0.59119635 | 7.99E-09 |
| Itfg1 | -0.590906005 | 2.73E-08 |
| Emc2 | -0.590867526 | 2.55E-08 |
| Thap2 | -0.590271336 | 9.19E-05 |
| Smim13 | -0.590034413 | 2.55E-08 |
| Acss3 | -0.589732109 | 0.002135825 |
| Mfsd4a | -0.58924107 | 3.73E-05 |
| Zfp953 | -0.589133282 | 0.002558673 |
| Nfib | -0.589002259 | 7.44E-05 |
| Dr1 | -0.588425438 | 2.89E-05 |
| Snx13 | -0.588351709 | 4.33E-09 |
| Rab5a | -0.587963128 | 1.31E-06 |
| Rock1 | -0.587659462 | 0.000267612 |
| Fam92a | -0.587513698 | 7.97E-05 |
| Tmem161b | -0.586969243 | 4.95E-06 |
| Fundc2 | -0.586549013 | 3.75E-08 |
| Tfg | -0.586459162 | 4.63E-07 |
| Eea1 | -0.586147429 | 2.97E-06 |
| Tyw3 | -0.585892424 | 0.000384199 |
| Fam57b | -0.585695047 | 0.001977474 |
| Pfn2 | -0.58540881 | 8.34E-07 |
| Acsl3 | -0.58540086 | 0.001483422 |
| Fam179b | -0.58420069 | 1.34E-06 |
| Cpeb3 | -0.583139741 | 1.75E-05 |
| Mpv17l | -0.582929566 | 0.033745318 |
| 5730455P16Rik | -0.581943489 | 8.54E-07 |
| Hdgfrp3 | -0.581846002 | 0.000369866 |
| 2210408I21Rik | -0.581234043 | 0.008012193 |
| Mid2 | -0.581045649 | 2.33E-05 |
| Efemp1 | -0.581025025 | 0.000261845 |
| Rhoq | -0.580799473 | 1.21E-08 |
| Polk | -0.580569331 | 2.01E-05 |
| Homer2 | -0.579951395 | 3.77E-05 |
| Ecm2 | -0.579135534 | 1.00E-05 |
| Chic1 | -0.57876632 | 0.015733703 |
| Fam161b | -0.578214702 | 0.003807726 |
| Slc16a12 | -0.577884956 | 0.029797874 |
| Zbtb41 | -0.577867928 | 3.01E-05 |
| Tmtc3 | -0.577437569 | 3.22E-05 |
| Psd3 | -0.57716765 | 1.97E-06 |
| Eif4e | -0.576920493 | 5.99E-07 |
| Has1 | -0.576834112 | 0.028841961 |
| Kyat3 | -0.576008175 | 0.000399248 |
| Uggt2 | -0.575320192 | 0.000216292 |
| Zfp933 | -0.574944379 | 8.95E-06 |
| Mum1l1 | -0.574355598 | 0.000388025 |
| Ppm1k | -0.574164943 | 8.50E-05 |
| Asah2 | -0.573816802 | 0.000148427 |
| Laptm4b | -0.573585038 | 2.61E-06 |
| Zfp748 | -0.573504647 | 0.000334896 |
| Dlat | -0.573338758 | 6.93E-07 |
| AI597479 | -0.572910767 | 1.82E-07 |
| Uchl4 | -0.572844136 | 0.002453485 |
| Usp9x | -0.572700927 | 2.88E-09 |
| Camk2d | -0.572660942 | 2.75E-06 |
| Ift74 | -0.572657881 | 0.000821245 |
| Lgalsl | -0.572628019 | 2.37E-07 |
| Dnajc28 | -0.572445065 | 2.13E-07 |
| Hspa13 | -0.572430449 | 7.74E-06 |
| Crebrf | -0.572257941 | 9.88E-06 |
| Casd1 | -0.572094209 | 8.57E-07 |
| Pak1 | -0.571733863 | 1.21E-06 |
| Trps1 | -0.571718267 | 0.000433503 |
| Gem | -0.571199148 | 0.000960808 |
| Adora2b | -0.571189353 | 0.027935856 |
| Epm2aip1 | -0.570796062 | 7.90E-10 |
| Pten | -0.570525249 | 5.09E-05 |
| Pdhx | -0.570380403 | 4.40E-07 |
| Mettl15 | -0.569585913 | 0.000355483 |
| Prkcq | -0.569386438 | 4.24E-06 |
| Sacm1l | -0.569368494 | 2.56E-07 |
| Tlr8 | -0.569116779 | 0.001830413 |
| Apool | -0.568601963 | 2.12E-07 |
| Ktn1 | -0.568226465 | 7.21E-06 |
| Tmem41b | -0.56799902 | 6.15E-06 |
| 1700021F05Rik | -0.567872653 | 8.63E-06 |
| Marcks | -0.567782928 | 1.87E-05 |
| Cobll1 | -0.567230913 | 0.000564347 |
| Serpine1 | -0.566701807 | 0.000787495 |
| Slc2a13 | -0.566637404 | 0.005296001 |
| Xiap | -0.566570434 | 3.52E-05 |
| Sorbs1 | -0.566138872 | 1.88E-07 |
| Intu | -0.565783595 | 0.000173224 |
| Angpt1 | -0.565485301 | 4.72E-05 |
| Fam180a | -0.565399885 | 0.012542112 |
| Scml2 | -0.565346571 | 0.007573374 |
| Ttc32 | -0.565256388 | 2.78E-05 |
| Denr | -0.565078033 | 1.64E-05 |
| Tmem56 | -0.564653814 | 2.08E-08 |
| Ppp2r3c | -0.56452803 | 1.26E-07 |
| Luc7l2 | -0.564393889 | 6.90E-05 |
| Pde3b | -0.56437561 | 0.001163436 |
| Usp28 | -0.564082855 | 2.71E-08 |
| Nkiras1 | -0.563865368 | 4.47E-05 |
| Il7r | -0.563789245 | 0.002706389 |
| Fhod3 | -0.56373698 | 3.98E-06 |
| Eya4 | -0.5626642 | 9.71E-05 |
| Ppp3r1 | -0.56248042 | 1.83E-08 |
| Zbtb26 | -0.56245117 | 2.59E-05 |
| Klhl23 | -0.562364464 | 2.83E-05 |
| Trpm3 | -0.562056242 | 0.036326436 |
| Mcts2 | -0.561875561 | 0.000956364 |
| Arl5b | -0.561518724 | 5.44E-05 |
| Elmod2 | -0.561467782 | 0.000146982 |
| Lpgat1 | -0.561458104 | 3.33E-09 |
| Prkab2 | -0.561452201 | 2.69E-05 |
| Zfp788 | -0.560902725 | 1.75E-05 |
| Agmo | -0.560847602 | 0.002598412 |
| Parm1 | -0.560777324 | 0.001198954 |
| Exph5 | -0.560526276 | 0.040087589 |
| Tex15 | -0.560522919 | 0.004442281 |
| Kcnc2 | -0.560303789 | 0.036951594 |
| Tarsl2 | -0.560274118 | 8.01E-07 |
| Adk | -0.560173462 | 1.35E-07 |
| Rpgr | -0.559991589 | 0.003845363 |
| Bend7 | -0.558962143 | 0.010693706 |
| Ces1d | -0.558489764 | 0.002118464 |
| Noct | -0.558301079 | 8.41E-07 |
| Klhl28 | -0.557293414 | 0.001504123 |
| Rtn4ip1 | -0.556965599 | 1.02E-06 |
| Gadd45g | -0.556806703 | 0.021887514 |
| Syncrip | -0.556323669 | 1.01E-05 |
| Khdrbs3 | -0.556161575 | 0.000224054 |
| Ola1 | -0.555982526 | 2.83E-06 |
| Depdc1a | -0.555615457 | 0.000797786 |
| Egr2 | -0.554642273 | 0.013048563 |
| Zfp760 | -0.554253557 | 0.002103759 |
| Sccpdh | -0.554168687 | 8.48E-06 |
| Moxd1 | -0.553542536 | 0.032949764 |
| Cul2 | -0.552654637 | 1.05E-06 |
| Rapgef5 | -0.552510453 | 0.000449599 |
| Golga7b | -0.5524495 | 0.037853133 |
| Papolg | -0.552180414 | 0.000164852 |
| Zscan30 | -0.551786113 | 0.005939598 |
| Arl13b | -0.551705075 | 0.000175017 |
| Mrpl1 | -0.551399245 | 8.12E-08 |
| Atp5f1 | -0.551356467 | 7.57E-07 |
| Dhrs9 | -0.55120182 | 0.005854049 |
| Tm9sf3 | -0.551107489 | 1.01E-06 |
| Tbc1d32 | -0.551072762 | 0.002856662 |
| Wfs1 | -0.551018556 | 1.50E-05 |
| Lrrc58 | -0.550501107 | 8.52E-06 |
| Trappc2 | -0.550278685 | 4.93E-06 |
| Phkb | -0.550137498 | 0.000111391 |
| Cops3 | -0.549703569 | 7.70E-07 |
| Csgalnact2 | -0.549588905 | 4.91E-05 |
| Hif3a | -0.549482035 | 0.024055738 |
| D16Ertd472e | -0.548840329 | 0.00126619 |
| Rmdn1 | -0.548570842 | 2.75E-07 |
| Dtna | -0.54836722 | 1.50E-05 |
| Hrasls | -0.548183007 | 0.000233504 |
| Zdhhc23 | -0.546580218 | 0.011239283 |
| Ppp1r9a | -0.546185159 | 0.001040308 |
| Zfp189 | -0.545504188 | 0.000330731 |
| Tceb1 | -0.545457736 | 1.29E-06 |
| Kpna1 | -0.545438063 | 1.43E-07 |
| Sncg | -0.545356673 | 0.012462907 |
| Tstd3 | -0.545346175 | 2.25E-06 |
| Acer2 | -0.544659832 | 0.012627236 |
| Fbxl5 | -0.544266654 | 1.84E-05 |
| Zfp62 | -0.544120839 | 2.09E-05 |
| Plod2 | -0.543993509 | 0.013467049 |
| Akr1c14 | -0.543946414 | 0.003489527 |
| Fam134b | -0.54376038 | 0.001588353 |
| Zfp229 | -0.543440627 | 0.000693501 |
| Tbc1d8b | -0.543235442 | 0.000105776 |
| Ergic2 | -0.543054154 | 2.84E-05 |
| Sec23a | -0.542275806 | 3.51E-06 |
| Got1 | -0.542139312 | 1.64E-05 |
| Zfp397 | -0.541853746 | 1.93E-06 |
| Tfb1m | -0.541751064 | 0.000101224 |
| Stk17b | -0.541650551 | 0.001781216 |
| Enox2 | -0.541638443 | 0.000706697 |
| Rnf115 | -0.540823538 | 1.24E-07 |
| Gprc5a | -0.540494356 | 0.012986625 |
| Ssr3 | -0.54046 | 2.02E-05 |
| Scn2a1 | -0.540458395 | 0.01633984 |
| Pibf1 | -0.540416463 | 8.76E-05 |
| Zfp24 | -0.54007316 | 3.44E-06 |
| Sirt1 | -0.539553088 | 0.000210682 |
| Pcp4l1 | -0.539033726 | 0.000510161 |
| Pdp1 | -0.538988948 | 0.000103297 |
| Fastkd2 | -0.538611179 | 9.16E-06 |
| Hrc | -0.538459274 | 0.000273553 |
| Atpaf1 | -0.538201094 | 2.62E-05 |
| Gm37240 | -0.537517523 | 0.000282385 |
| Tmem170b | -0.537371553 | 1.85E-05 |
| Hes1 | -0.537171647 | 0.042046136 |
| Gabpa | -0.537107993 | 8.02E-06 |
| Clec3a | -0.537089561 | 0.025865282 |
| Tpd52l1 | -0.536768776 | 0.000202649 |
| Aldh6a1 | -0.535802397 | 1.69E-06 |
| Shoc2 | -0.535638811 | 9.74E-05 |
| Gpr141 | -0.535558358 | 0.009102387 |
| Stag2 | -0.535539277 | 0.000121642 |
| Zc2hc1a | -0.535223924 | 0.00029541 |
| Akap6 | -0.535026591 | 8.92E-06 |
| Mrpl32 | -0.53482495 | 0.000184216 |
| Cd69 | -0.534464508 | 0.01471781 |
| Nudcd1 | -0.534400355 | 9.77E-05 |
| Klhl9 | -0.534210814 | 7.17E-06 |
| Srek1ip1 | -0.534208137 | 5.41E-05 |
| Zfp420 | -0.533727818 | 0.004939048 |
| Magohb | -0.533724382 | 0.001511903 |
| Cript | -0.53300241 | 2.84E-07 |
| Usmg5 | -0.532625971 | 3.68E-06 |
| Dpf1 | -0.532458843 | 0.027820559 |
| Zfp11 | -0.532241236 | 0.003249882 |
| Msrb3 | -0.532128111 | 1.12E-05 |
| Ptgr2 | -0.531977074 | 1.46E-07 |
| Ttc26 | -0.531476372 | 0.018550522 |
| Jrkl | -0.531347814 | 0.004096452 |
| Bnip3 | -0.531218085 | 2.83E-06 |
| Tnks2 | -0.531214453 | 1.15E-07 |
| Eif4a2 | -0.530151788 | 3.73E-07 |
| Med13 | -0.529257587 | 2.68E-05 |
| Ear6 | -0.529062887 | 0.001262767 |
| Zranb2 | -0.528897915 | 1.96E-05 |
| Asb5 | -0.528755375 | 7.86E-05 |
| Aasdhppt | -0.528681313 | 7.22E-06 |
| Klhl38 | -0.528266084 | 5.04E-05 |
| Rbms3 | -0.52804799 | 0.000428966 |
| Gphn | -0.527614077 | 9.32E-05 |
| Cth | -0.527137651 | 0.036380817 |
| Zfp712 | -0.527006371 | 0.010992472 |
| Ebag9 | -0.526721682 | 0.000136387 |
| Qser1 | -0.526535379 | 0.00032361 |
| G2e3 | -0.526242314 | 0.000653287 |
| Rbm41 | -0.526156762 | 0.000455635 |
| Appl1 | -0.526129322 | 8.68E-05 |
| Creb1 | -0.525814591 | 0.000479095 |
| Rnf103 | -0.525627472 | 7.12E-07 |
| G3bp2 | -0.525489989 | 1.17E-06 |
| Tmem117 | -0.525060627 | 3.35E-05 |
| Svip | -0.52503685 | 1.08E-06 |
| Hspa9 | -0.524793331 | 4.15E-08 |
| Abi2 | -0.523789289 | 0.000494524 |
| Crem | -0.523643843 | 8.83E-05 |
| Nabp1 | -0.523641771 | 2.47E-05 |
| Chrne | -0.52306614 | 0.001436601 |
| Il13ra1 | -0.522825163 | 0.001398831 |
| Ikzf5 | -0.522774874 | 0.000155591 |
| Bet1 | -0.522208039 | 4.52E-05 |
| Nt5c1a | -0.521998762 | 0.008784311 |
| Eif5a2 | -0.521862313 | 0.012306232 |
| Crim1 | -0.521354853 | 4.39E-05 |
| Pmpcb | -0.521305373 | 1.27E-07 |
| Eif1a | -0.521129574 | 0.00030864 |
| Gfm1 | -0.520052619 | 1.15E-07 |
| Atxn2 | -0.520041037 | 6.05E-05 |
| B230118H07Rik | -0.519972703 | 5.63E-05 |
| Akap7 | -0.51989934 | 0.001461225 |
| Ireb2 | -0.519374652 | 5.33E-07 |
| Bcl6b | -0.51925485 | 0.00930458 |
| Zkscan8 | -0.518586989 | 9.84E-05 |
| Xpo1 | -0.518467628 | 4.65E-05 |
| Glul | -0.518077308 | 3.51E-06 |
| Csrp3 | -0.517913119 | 0.005428722 |
| Ret | -0.517905237 | 0.000453122 |
| Fam151b | -0.51775596 | 0.017169665 |
| Wasl | -0.517694404 | 0.000119525 |
| Slc35a3 | -0.517590215 | 0.000764613 |
| Stxbp6 | -0.516928522 | 0.000552196 |
| BC003331 | -0.516825065 | 1.42E-05 |
| Arhgap29 | -0.516641577 | 3.73E-05 |
| Zyg11b | -0.515771225 | 1.11E-07 |
| Dhx36 | -0.515727532 | 5.42E-06 |
| Gm527 | -0.515724808 | 0.026985916 |
| Txlnb | -0.515630738 | 4.80E-05 |
| Prrg3 | -0.515417627 | 0.001396569 |
| Camk2b | -0.515238587 | 0.000704244 |
| Arhgef9 | -0.514996742 | 0.000740929 |
| Trim16 | -0.514987951 | 3.04E-07 |
| Fibin | -0.514744968 | 3.73E-05 |
| Zdhhc21 | -0.513993909 | 1.76E-05 |
| BC030336 | -0.51382902 | 3.45E-05 |
| Fxr1 | -0.513476477 | 4.77E-05 |
| Gstm2 | -0.513397565 | 0.002265646 |
| Magix | -0.513268592 | 0.008470059 |
| Atp13a3 | -0.513140648 | 7.68E-05 |
| Uba3 | -0.512680858 | 2.01E-06 |
| Arhgap12 | -0.512645504 | 9.44E-06 |
| Ndufs1 | -0.512461751 | 1.23E-06 |
| Ddx3x | -0.512324012 | 1.81E-06 |
| Pstpip2 | -0.511497658 | 7.52E-05 |
| Ehbp1 | -0.511464028 | 0.000148905 |
| Msantd4 | -0.510767028 | 1.27E-05 |
| Ctbs | -0.510449343 | 0.004312742 |
| Rc3h1 | -0.510360221 | 1.41E-05 |
| Capza2 | -0.510313111 | 1.59E-06 |
| Dpy19l4 | -0.510298555 | 1.55E-05 |
| Mrap | -0.510284186 | 0.007447734 |
| Srsf10 | -0.510273078 | 0.000455635 |
| Clock | -0.510238743 | 4.26E-05 |
| Nova1 | -0.510227338 | 0.00519481 |
| Sorbs2 | -0.510168919 | 0.000400453 |
| Ppp1r2 | -0.510095318 | 5.71E-07 |
| Pank3 | -0.509957957 | 6.05E-05 |
| Ccdc181 | -0.50959026 | 0.000725303 |
| Uqcrb | -0.509550874 | 1.62E-05 |
| Deptor | -0.509507588 | 0.005092264 |
| Gpcpd1 | -0.509405842 | 1.50E-05 |
| AI987944 | -0.509272541 | 0.00010831 |
| Wdr44 | -0.509006514 | 6.48E-05 |
| Zfp91 | -0.508646728 | 1.22E-07 |
| Fubp1 | -0.508483656 | 0.00222742 |
| Ttll7 | -0.508112493 | 0.000699141 |
| Vps13a | -0.507902131 | 1.50E-05 |
| 0610010F05Rik | -0.507788819 | 2.37E-06 |
| Kbtbd8 | -0.507784909 | 0.006290255 |
| Zfp799 | -0.507767911 | 0.001806549 |
| Cxadr | -0.507582814 | 0.013608595 |
| Mrpl35 | -0.507451635 | 0.000130724 |
| Pdcl | -0.507283523 | 0.000136393 |
| Ciart | -0.507185599 | 0.018380051 |
| Crls1 | -0.507051778 | 0.000492827 |
| Cpne8 | -0.506915648 | 0.007376359 |
| Hspb7 | -0.506117657 | 0.000774957 |
| Pde4b | -0.50581381 | 6.09E-06 |
| Rtn4 | -0.505763237 | 6.93E-05 |
| Hif1a | -0.505403913 | 0.004127194 |
| Mospd1 | -0.505137057 | 0.000185671 |
| Vamp3 | -0.504880144 | 9.27E-06 |
| Tlr3 | -0.504787918 | 0.003677542 |
| Zfp260 | -0.504766544 | 9.89E-06 |
| Tceal1 | -0.504603198 | 0.00468934 |
| Apol6 | -0.504531593 | 0.000201486 |
| Fam208a | -0.504283048 | 0.011448675 |
| Map4k3 | -0.504218688 | 0.000132924 |
| Sumo2 | -0.504150457 | 9.47E-05 |
| Sucla2 | -0.503777906 | 1.97E-06 |
| Sp3 | -0.503690515 | 0.006479485 |
| Sppl2a | -0.503685744 | 9.43E-06 |
| Fam135a | -0.503599487 | 0.00037263 |
| Celf2 | -0.503474604 | 0.000369866 |
| Nfia | -0.503367663 | 0.000344885 |
| Coq7 | -0.503294082 | 0.0002208 |
| Oxr1 | -0.50310004 | 7.38E-06 |
| Cul4b | -0.502955294 | 0.00011461 |
| Osbpl8 | -0.502621287 | 0.002429052 |
| Myh2 | -0.50225185 | 5.12E-05 |
| Tspan2 | -0.502210468 | 0.000141038 |
| Lmbrd2 | -0.502116523 | 0.002527211 |
| Crbn | -0.501889771 | 1.14E-06 |
| Olfr558 | -0.501556137 | 0.002484915 |
| Gbp7 | -0.501187199 | 0.001807067 |
| Mrpl42 | -0.500855384 | 0.00030562 |
| Katnbl1 | -0.500800437 | 0.001525333 |
| Lpin3 | -0.500188041 | 0.00493395 |
| Zfp871 | -0.500129616 | 0.000119849 |
| Abhd17b | -0.500117401 | 0.000421427 |
| Pcnp | -0.500107964 | 0.001359407 |
| Tmem70 | -0.50010043 | 2.12E-05 |

**Supplementary Table 3. Gene ontology of upregulated genes**

**Supplementary Table 4. Gene ontology of downregulated genes**
